# Metabolites enhance innate resistance to human *Mycobacterium tuberculosis* infection

**DOI:** 10.1172/jci.insight.152357

**Published:** 2022-11-22

**Authors:** Deepak Tripathi, Kamakshi Prudhula Devalraju, Venkata Sanjeev Kumar Neela, Tanmoy Mukherjee, Padmaja Paidipally, Rajesh Kumar Radhakrishnan, Igor Dozmorov, Abhinav Vankayalapati, Mohammad Soheb Ansari, Varalakshmi Mallidi, Anvesh Kumar Bogam, Karan P. Singh, Buka Samten, Vijaya Lakshmi Valluri, Ramakrishna Vankayalapati

**Affiliations:** 1 Department of Pulmonary Immunology and Center for Biomedical Research, School of Community and Rural Health, University of Texas Health Science Center, Tyler, Texas, USA.; 2 Immunology and Molecular Biology Department, Bhagwan Mahavir Medical Research Centre, Hyderabad, India.; 3 Department of Immunology, UT Southwestern Medical Center, Dallas, Texas, USA.; 4 Department of Epidemiology and Biostatistics, School of Community and Rural Health, University of Texas Health Science Center, Tyler, Texas, USA.

**Keywords:** Immunology, Tuberculosis

## Abstract

To determine the mechanisms that mediate resistance to *Mycobacterium tuberculosis* (*M. tuberculosis*) infection in household contacts (HHCs) of patients with tuberculosis (TB), we followed 452 latent TB infection–negative (LTBI^–^) HHCs for 2 years. Those who remained LTBI^–^ throughout the study were identified as nonconverters. At baseline, nonconverters had a higher percentage of CD14^+^ and CD3^–^CD56^+^CD27^+^CCR7^+^ memory-like natural killer (NK) cells. Using a whole-transcriptome and metabolomic approach, we identified deoxycorticosterone acetate as a metabolite with elevated concentrations in the plasma of nonconverters, and further studies showed that this metabolite enhanced glycolytic ATP flux in macrophages and restricted *M. tuberculosis* growth by enhancing antimicrobial peptide production through the expression of the surface receptor sialic acid binding Ig-like lectin–14. Another metabolite, 4-hydroxypyridine, from the plasma of nonconverters significantly enhanced the expansion of memory-like NK cells. Our findings demonstrate that increased levels of specific metabolites can regulate innate resistance against *M. tuberculosis* infection in HHCs of patients with TB who never develop LTBI or active TB.

## Introduction

*Mycobacterium tuberculosis (M. tuberculosis*) causes almost 1.4 million deaths annually (1). After exposure to a patient with tuberculosis (TB), 50% of household contacts (HHCs) develop latent TB infection (LTBI) and remain healthy, while 10% of LTBI^+^ individuals eventually develop TB (2). However, some HHCs of patients with TB remain healthy and have negative tuberculin skin tests, indicating that they do not have LTBI (3). Similarly, some doctors, nurses, and other health care workers who have worked with TB patients for many years never develop LTBI or active TB (4, 5). To develop better methods to prevent and treat TB, we must understand how it is that many contacts never develop LTBI or active TB. Harnessing these immune mechanisms is critical in combating LTBI and TB.

Most studies on immunity to TB have focused on adaptive immunity (6). However, several studies suggest that immune responses other than T cells play a significant role in protection against TB. Polymorphisms in Toll interacting protein and Unc51-like kinase 1 are associated with LTBI susceptibility (7).

Compared with LTBI^+^ individuals, individuals who never develop latent infection (nonconverters) display enhanced antibody avidity and distinct *M. tuberculosis*–specific IgG Fc profiles (8). Monocytes from nonconverters respond differently, and transcriptional responses are distinct during ex vivo *M. tuberculosis* infection (9). LTBI^–^ individuals who remain LTBI^–^ after long-term follow-up have strong humoral and noncanonical cellular immunity to *M. tuberculosis* (8), suggesting that a deeper understanding of the immune responses of LTBI^–^ contacts of patients with TB using established cohorts is essential.

In the present study, we used an unbiased approach to determine underlying immune mechanisms in HHCs of patients with TB who never develop LTBI or active TB despite significant exposure. We characterized the phenotype and function of various immune cells in a large group of LTBI^–^ HHCs (nonconverters) of patients with TB (a total of 452 individuals). We performed these measures at baseline (0 months) and follow-up (24 months) while monitoring the participants for conversion to LTBI. By following a large cohort of HHCs of patients with TB and performing transcriptional and metabolomic studies, we determined whether resistance to *M. tuberculosis* infection is preceded by specific changes in the transcriptional profile. We also determined whether metabolites regulate macrophage metabolism and function to restrict *M. tuberculosis* growth.

## Results

### Prevalence of nonconverters in the HHCs of patients with TB.

**Figure 1:**
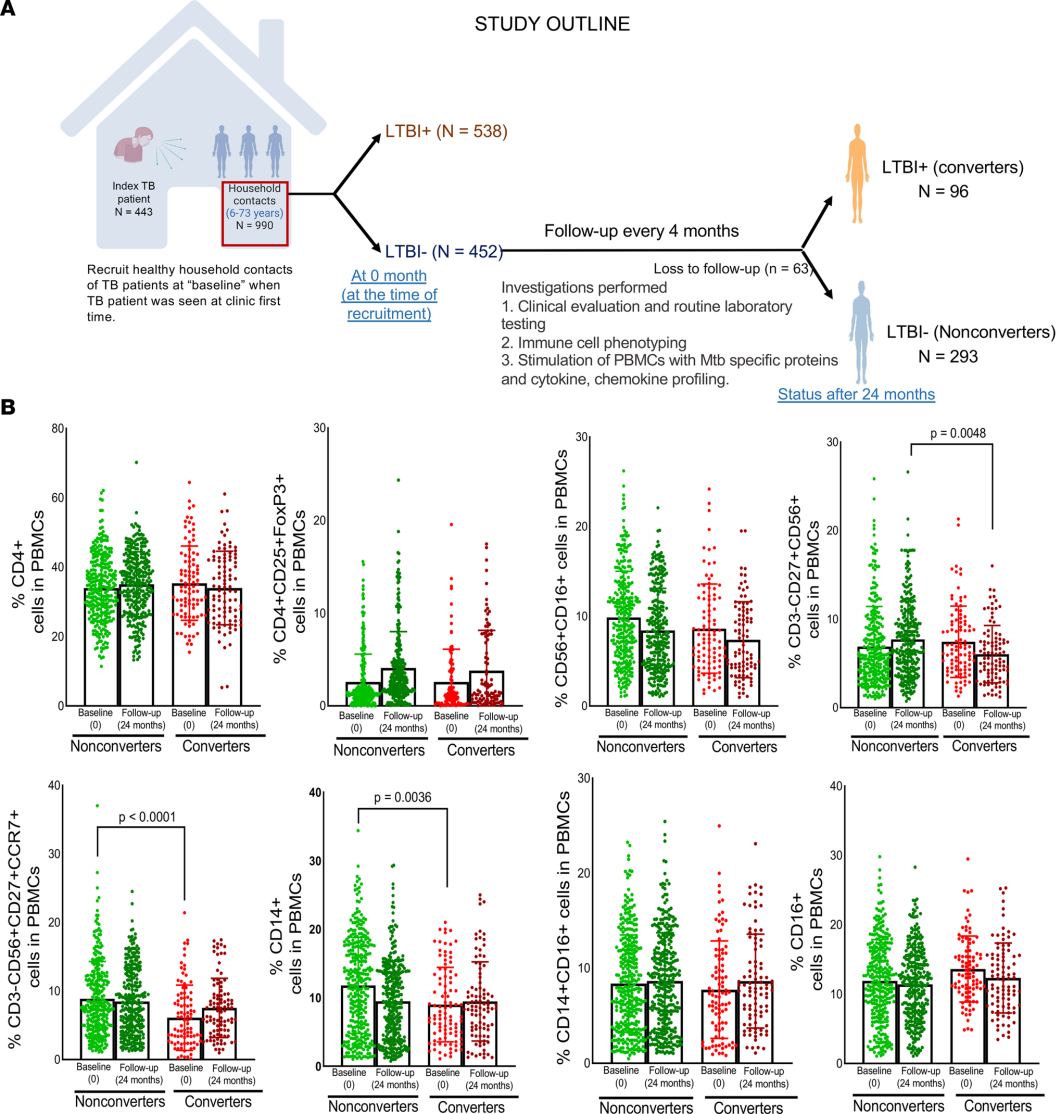
Study design and conversion of LTBI^–^ individuals to LTBI^+^ individuals in a cohort of HHCs of patients with TB. (**A**) Schematic representation of the experimental design and conversion of LTBI^–^ HHCs (*n* = 452) of patients with TB into LTBI^+^ (converters) and remaining LTBI^–^ (nonconverters) at 24 months of follow-up. (**B**) Immune cell populations in the peripheral blood mononuclear cells (PBMCs) of *M*. *tuberculosis*–exposed HHCs of patients with TB. PBMCs were isolated from age-matched, epidemiological risk–matched, healthy (no comorbid conditions and not on any immunosuppressive drugs) converters (*n* = 96) and nonconverters (*n* = 293) at baseline (during the enrollment of the study, when all participants were healthy and LTBI^–^, and after 24 months), and the percentages of CD4^+^, CD4^+^CD25^+^FoxP3^+^, CD56^+^CD16^+^, CD3^–^CD56^+^CD27^+^, CD3^–^CD56^+^CD27^+^CCR7^+^, CD14^+^CD16^+^, and CD16^+^ cells were determined by flow cytometry. The *P* values were determined by repeated measures mixed effects ANOVA followed by post hoc Tukey’s multiple comparisons test. Mean values, SDs, and *P* values are shown. Baseline, 0 months: at the time of recruitment; follow-up: 24 months after enrollment in the study.

At baseline, among 990 HHCs, 538 (54.3%) were positive and 452 (45.6%) were negative for LTBI by the IFN-γ release assay (IGRA) as described in the Methods (Supplemental Table 1; supplemental material available online with this article; https://doi.org/10.1172/jci.insight.152357DS1). We followed these 452 LTBI^–^ HHCs for 2 years at 4-month intervals (study plan is shown in Figure 1A). Exposure to index patients was determined following the criteria mentioned in previous studies (10–14) and in the Methods section. Among the 452 LTBI^–^ HHCs, 96 (21.2%) became IGRA^+^ (converters) over 2 years of follow-up. Despite similar exposure levels, 293 (64.8%) HHCs remained IGRA^–^ (nonconverters) until the end of the study (Table 1 and Supplemental Data 1). As shown in Supplemental Data 1, all (*n* = 96) converters consistently produced IFN-γ in response to *M. tuberculosis* antigens. The demographic details of converters and nonconverters are shown in Table 1 and Supplemental Table 1.

### Immune cell phenotypes of converters and nonconverters.

The innate immune response is critical to clear *M. tuberculosis* infection and may play an important role in preventing LTBI conversion (15–18). We determined various immune cell populations at baseline and during the follow-up visits (24 months) in freshly isolated peripheral blood mononuclear cells (PBMCs) of age- and sex-matched nonconverters (*n* = 293) and converters (*n* = 96). At baseline, in fresh PBMCs of nonconverters, the percentages of CD14^+^ and CD3^–^CD56^+^CD27^+^CCR7^+^ memory-like NK cells were significantly higher than those of converters (Figure 1B and Supplemental Figure 1). No significant differences in CD4^+^, CD16^+^, CD14^+^CD16^+^, CD16^+^CD56^+^, or CD4^+^CD25^+^FoxP3^+^ cells were found in the PBMCs of nonconverters and converters (Figure 1B). As shown in Figure 1B, the percentage of memory-like NK cells significantly decreased in converters at follow-up. However, in nonconverters, the percentages of the cells remained the same throughout the study (Figure 1B).

### Cytokine and chemokine production by the PBMCs of converters and nonconverters in response to M. tuberculosis antigens.

**Figure 2:**
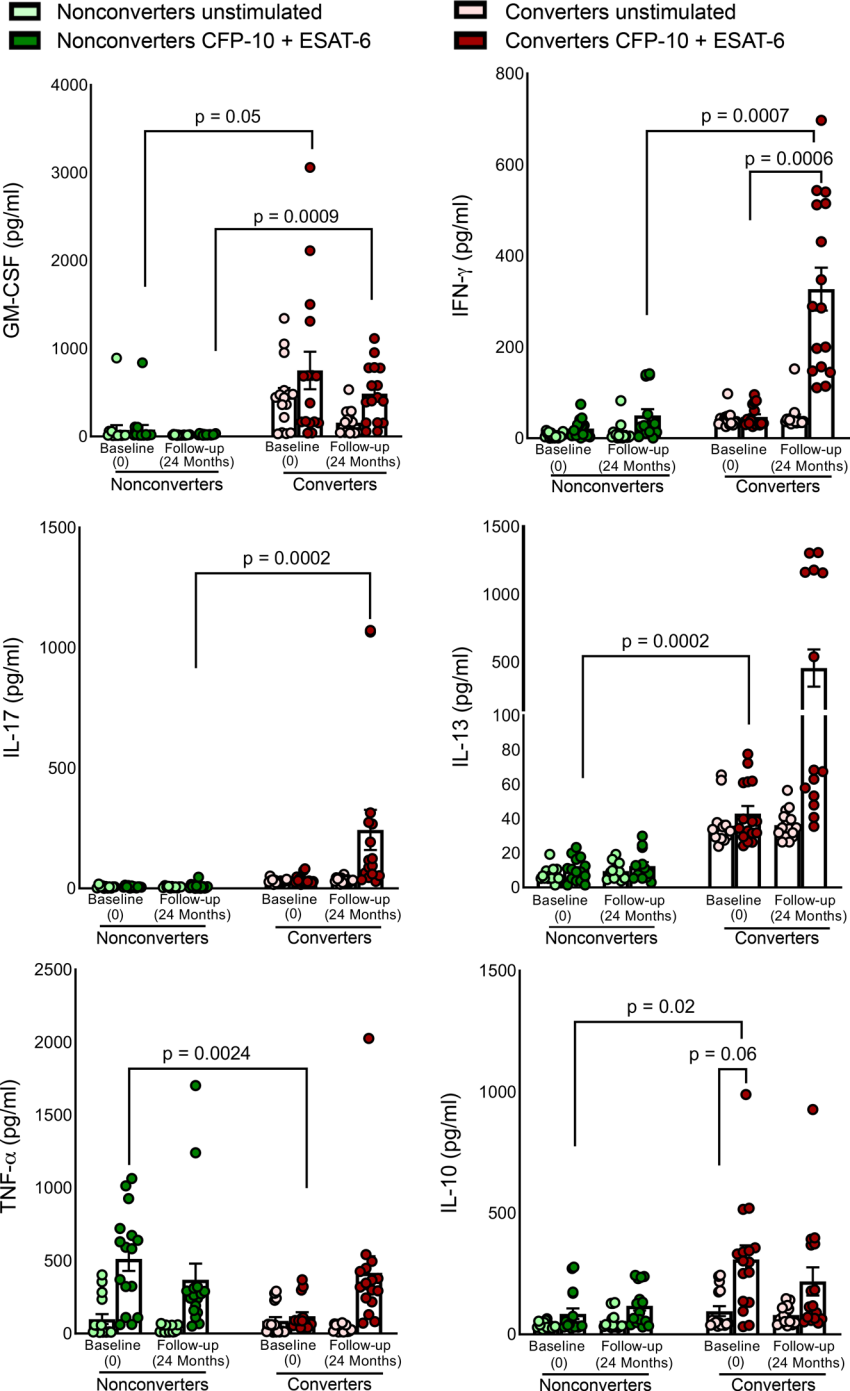
Cytokine and chemokine production by PBMCs of nonconverters and converters at baseline (0 time point) and during follow-up (after 24 months). PBMCs were isolated from age-matched, epidemiological risk–matched, healthy (no comorbid conditions and no immunosuppressive drugs) converters (*n* = 16) and nonconverters (*n* = 16) at baseline (during the enrollment of study, when all participants were healthy and LTBI^–^) and after 24 months and cultured with or without ESAT-6 and CFP-10 (10 μg/mL each), as described in the Methods section. After 96 hours, the culture supernatants were collected, and the levels of the various chemokines and cytokines were measured using a multiplex ELISA. The *P* values were derived using repeated measures mixed-effects ANOVA followed by post hoc Tukey’s multiple comparisons test. Mean values and SEM are shown.

Various cytokines and chemokines produced following *M. tuberculosis* exposure are crucial to the outcome of infection (19, 20). We determined cytokine and chemokine production by PBMCs of converters (*n* = 16) and nonconverters (*n* = 16) cultured in the presence and absence of 2 major *M. tuberculosis* antigens, ESAT-6 and CFP-10. Among the 34 cytokines and chemokines tested, as shown in Figure 2, at baseline, the production of TNF-α was higher in the culture supernatants of nonconverters than converters (*P* = 0.0024); however, during follow-up, no significant differences were observed. At baseline (all donors were negative for IFN-γ release), GM-CSF, IL-13, and IL-10 levels were significantly higher in the culture supernatants of converters than in those of nonconverters. At follow-up, IFN-γ (*P* = 0.0007) and IL-17 (*P* = 0.0002) levels were higher in converters than nonconverters (Figure 2 and Supplemental Figure 2).

### Nonconverters exhibit differential transcriptional signatures compared with converters.

**Figure 3:**
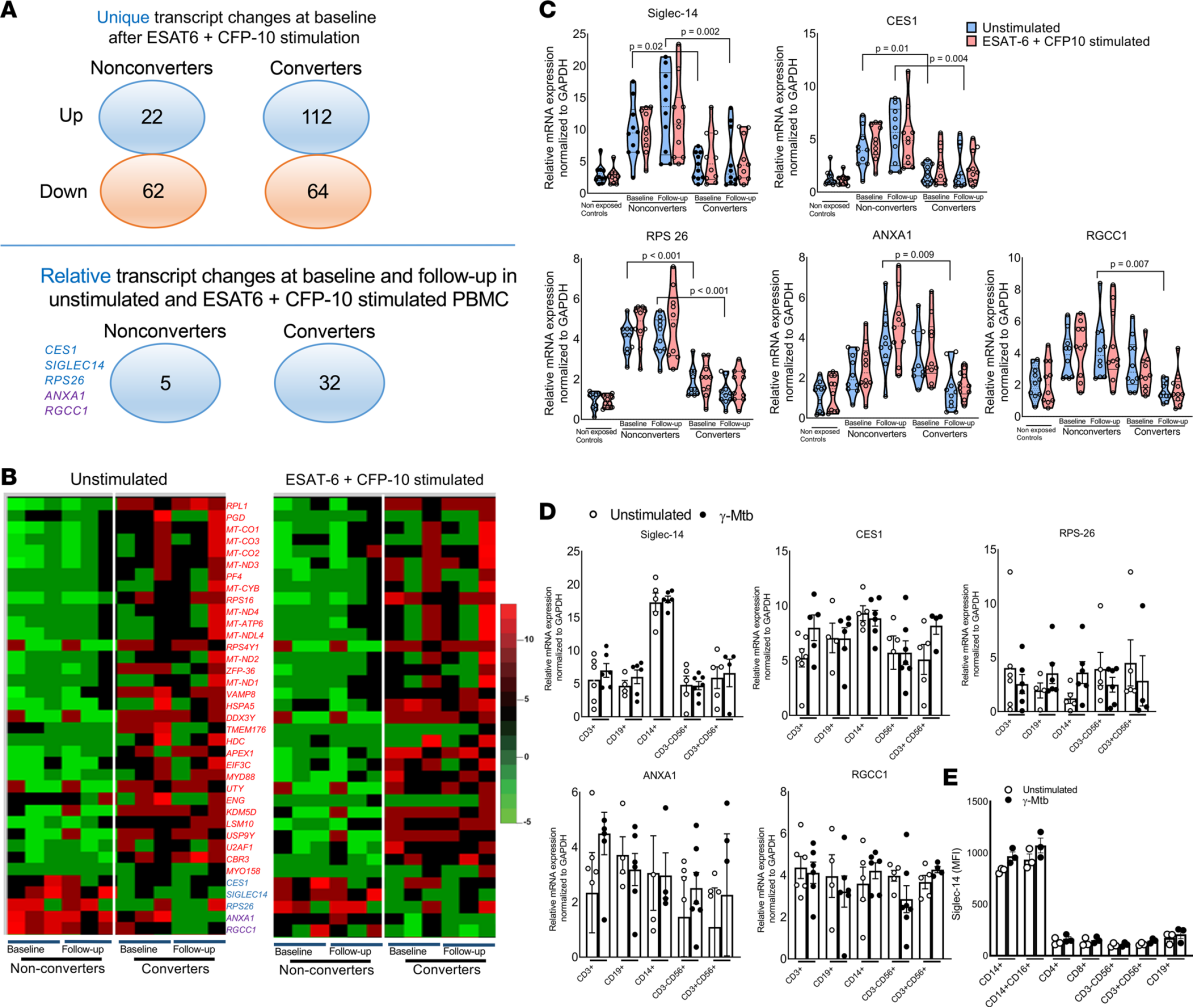
Whole-transcriptome sequencing analysis of ESAT-6– and CFP-10–cultured PBMCs from HHCs of patients with TB. (**A**) PBMCs were isolated from age-matched, epidemiological risk–matched, healthy (no comorbid conditions and no immunosuppressive drugs) nonconverters (*n* = 3) and converters (*n* = 3) at baseline (during study enrollment, when all participants were LTBI^–^) and after 24 months. Freshly isolated PBMCs were cultured with or without ESAT-6 and CFP-10 (10 μg/mL each), as described in the Methods section. After 96 hours, RNA was extracted, cDNA libraries were prepared, and whole-transcriptome sequencing was performed. The numbers of unique and relative transcript changes in unstimulated and ESAT-6 + CFP-10–stimulated PBMCs of converters and nonconverters at baseline and follow-up are shown. (**B**) A representative heatmap is shown. Transcripts differentially expressed in the PBMCs of LTBI^–^ compared with LTBI^+^ and active TB (*P* < 0.05, ANOVA). Diagram showing differentially expressed transcripts in nonconverters compared with converters. (**C**) PBMCs were obtained from nonconverters (*n* = 10) and converters (*n* = 10) at baseline and follow-up and from unexposed healthy controls (*n* = 10) and cultured in the presence of ESAT-6 + CFP-10 (10 μg/mL each), as described in the Methods section. After 96 hours, RNA was extracted, and the mRNA expression levels of Siglec-14, CES1, RPS-26, ANXA1, and RGCC1 were determined by quantitative real-time PCR. (**D**) PBMCs were obtained from healthy donors (*n* = 5) and cultured in the presence or absence of γ-*M*. *tuberculosis* (10 μg/mL). After 96 hours, various immune cell populations were sorted, and the relative mRNA expression levels of Siglec-14, CES1, RPS-26, ANXA1, and RGCC1 were determined by quantitative real-time PCR. (**E**) PBMCs were obtained from healthy donors (*n* = 3) and cultured in the presence or absence of γ-*M*. *tuberculosis* (10 μg/mL). After 96 hours, the expression of Siglec-14 in various immune populations was determined by flow cytometry. The *P* values were determined by 1-way ANOVA with Tukey’s multiple comparisons test. Mean values, SDs, and *P* values are shown.

Most transcriptional profiling studies in nonconverters and LTBI converters have used whole blood or PBMCs without *M. tuberculosis* antigen stimulation (21–29), suggesting the potential for interference with conditions other than *M. tuberculosis* exposure. Here, we determined the transcriptional signature of the above groups of donor PBMCs after *M. tuberculosis* antigen ESAT-6 and CFP-10 stimulation. We obtained freshly isolated PBMCs from converters (*n* = 3) and nonconverters (*n* = 3) at baseline and during follow-up and cultured them in the presence and absence of ESAT-6 and CFP-10 for 96 hours. The global gene expression pattern was determined using whole-transcriptome sequencing. An average of 75.6 million raw sequencing reads were generated, and after normalization and applying a threshold of ≥1 fragment per kilobase of transcript per million mapped reads, we detected approximately 15,529 genes per sample. To assess the reproducibility of our data, we calculated correlations across the biological replicates and found high correlations (Spearman’s correlation coefficient, mean *P* = 0.9707 ± 0.009), implying that the results were reproducible. To identify the common transcript changes in converters and nonconverters in response to *M. tuberculosis* antigen exposure at baseline, we compared the whole transcripts and found 140 upregulated transcripts and 106 downregulated transcripts in ESAT-6 + CFP-10–stimulated PBMCs from both groups of HHCs (*P* < 0.05, ANOVA, Tukey’s *t* test; Supplemental Figure 3 and Supplemental Data 2). As shown in Supplemental Figure 3, a total of 22 common transcripts, including some genes related to immune function, including IL-36γ, CCL3, CCL4, IL-20, MMP1, and TRAF4, were highly expressed at baseline in antigen-stimulated PBMCs of both converters and nonconverters. A total of 112 transcripts were highly expressed, and 64 transcripts were expressed at lower levels in ESAT-6 + CFP-10–stimulated PBMCs of converters than nonconverters. In contrast, 22 transcripts were highly expressed, and 62 transcripts were expressed at low levels in nonconverter PBMCs after *M. tuberculosis* antigen stimulation compared with converter PBMCs (Figure 3A and Supplemental Figure 4). Whole-transcriptome comparisons revealed that 32 unique protein-coding transcripts (red color) were significantly upregulated in unstimulated and ESAT-6 + CFP-10–stimulated PBMCs of the converters at baseline and during follow-up (Figure 3B). Three transcripts, carboxylesterase (CES) 1, sialic acid binding Ig-like lectin (Siglec) 14, and ribosomal protein (RPS) 26, had the highest relative expression in the PBMCs of nonconverters compared with those of converters at baseline and during follow-up, and 2 transcripts, regulator of the cell cycle (RGCC) 1 and annexin (ANXA) 1, had the highest relative expression during follow-up. Furthermore, we validated the transcriptome analysis data by real-time PCR analysis (Figure 3C). We also determined the expression of the above 5 molecules by purified monocytes, NK cells, and T cells isolated from control and γ-*M. tuberculosis*–stimulated PBMCs from healthy individuals. All 5 molecules were constitutively expressed by these cell populations (Figure 3, D and E). The monocyte population expressed a higher level of Siglec-14 than other cell populations (Figure 3, D and E, and Supplemental Figure 5A). However, the Siglec-14 expression level on CD14^+^ cells did not change with γ-*M. tuberculosis* stimulation (Figure 3E and Supplemental Figure 5B). In vitro differentiation of monocytes in the presence of human plasma changes the phenotype (30). Next, we determined the expression level of Siglec-14 on monocytes in the presence or absence of plasma from nonconverters and converters collected at baseline. We found that plasma from nonconverters (but not converters) enhanced Siglec-14 expression by γ-*M. tuberculosis*–cultured monocytes obtained from healthy LTBI^–^ nonhousehold contacts (Supplemental Figure 5C).

### Siglec-14 regulates M. tuberculosis growth in monocyte-derived macrophages.

**Figure 4:**
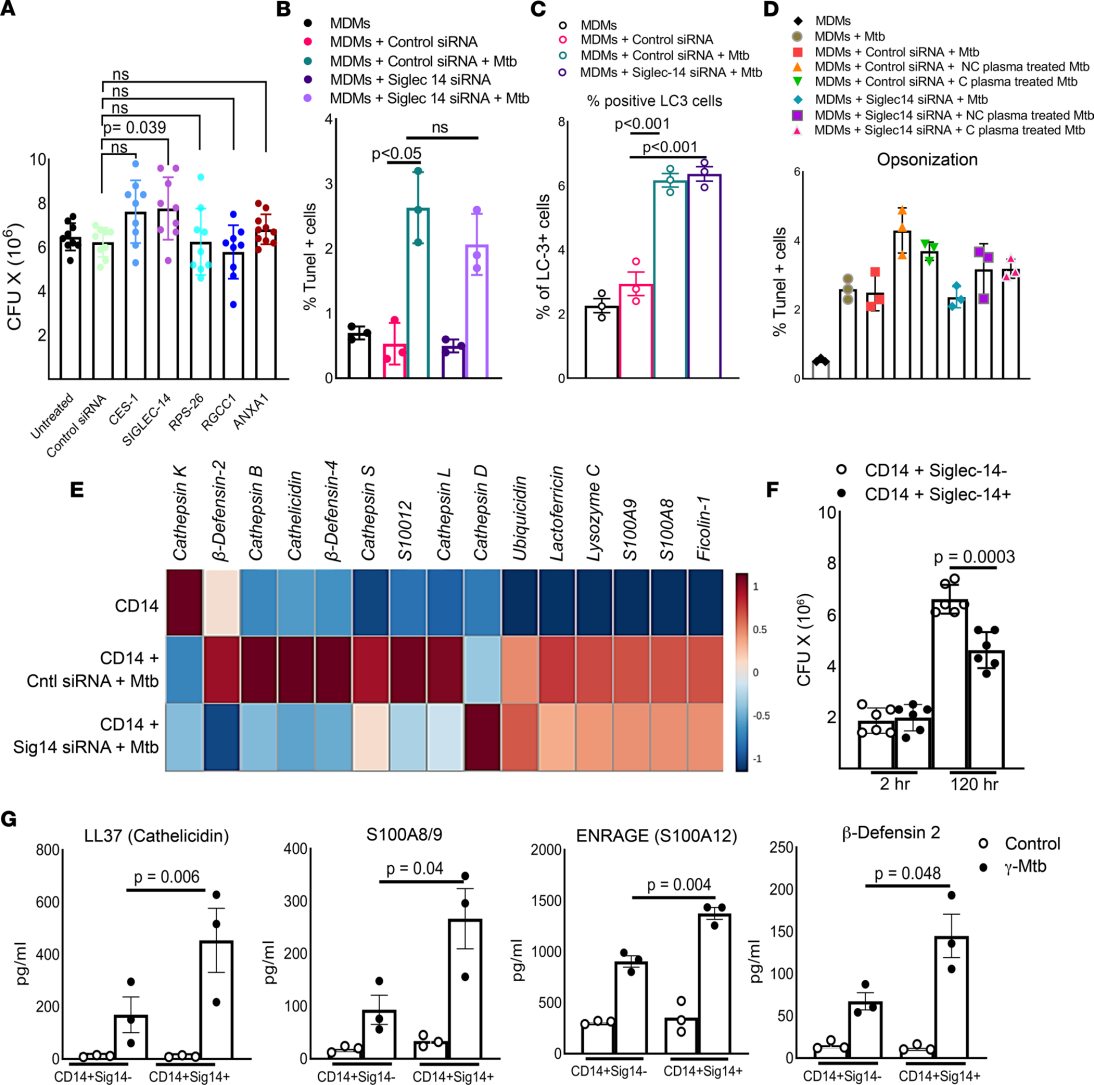
Siglec-14 reduces *M. tuberculosis* growth in MDMs through antimicrobial peptide production. MDMs from LTBI^–^ healthy donors were transfected with siRNA targeting CES-1, Siglec-14, RPS-26, RGCC1, and ANXA1 and control siRNA. The siRNA-transfected MDMs were infected with H37Rv at an MOI of 2.5. (**A**) After 5 days, the supernatant was aspirated, and the MDMs were lysed. The supernatant was centrifuged to pellet the bacteria, and the pellets were added to the cell lysates. The bacterial suspensions were ultrasonically dispersed, serially diluted, and plated in triplicate on 7H10 agar. The number of resultant colonies was counted after 3 weeks. The *P* values were determined by unpaired 2-tailed *t* test. The mean ± SD is shown. The means and SDs are shown for the number of CFUs per well. (**B**) The number of apoptotic MDMs was determined by flow cytometry. (**C**) The percentage of LC3^+^ MDMs was determined by flow cytometry. (**D**) Freshly prepared MDMs were infected with converter or nonconverter plasma opsonized or unopsonized *M*. *tuberculosis* H37Rv at an MOI of 2.5. The *P* values were determined by 1-way ANOVA with Tukey’s multiple comparisons test. Means, SDs, and *P* values are shown. (**E**) Control or Siglec-14 siRNA–transfected MDMs were infected with H37Rv at an MOI of 2.5. After 72 hours, RNA was isolated from MDMs, and a PCR array was performed for antimicrobial peptides. Data were normalized (*z* score) and centered using the Clustvis program. (**F**) CD14^+^Siglec-14^+^ and CD14^+^Siglec-14^–^ cells were magnetically sorted from the PBMCs of healthy donors (*n* = 6). Sorted cells were infected with *M*. *tuberculosis* H37Rv at an MOI of 2.5. At 2 hours and 5 days postinfection, the number of bacterial colonies was determined as outlined above. (**G**) Freshly isolated PBMCs from LTBI^–^ healthy donors (*n* = 3) were cultured in the presence or absence of γ-*M*. *tuberculosis*. After 72 hours, CD14^+^Siglec-14^+^ and CD14^+^Siglec-14^–^ cells were sorted through magnetic labeling, and RNA was isolated. Quantitative real-time PCR was performed to determine the mRNA expression level of antimicrobial peptides. The *P* values were determined by 1-way ANOVA with Tukey’s multiple comparisons test. Mean values, SDs, and *P* values are shown.

We determined the role of the above 5 molecules on *M. tuberculosis* growth in monocyte-derived macrophages (MDMs). MDMs from healthy individuals (*n* = 9–10) were isolated and transfected with siRNAs against CES1, Siglec-14, RPS26, RGCC1, and ANXA1. siRNA-transfected MDMs were infected with *M. tuberculosis* H37Rv at an MOI of 2.5. Control or other siRNA treatment did not affect the viability of MDMs (Supplemental Figure 6A). The silencing efficiency was confirmed by real-time PCR for all siRNAs using total RNA of siRNA-transfected MDMs (Supplemental Figure 6B). siRNA targeting CES1, Siglec-14, RPS26, RGCC1, and ANXA1 did not affect *M. tuberculosis* uptake compared with the control siRNA (Supplemental Figure 6C). As shown in Figure 4A, at 5 days postinfection, *M. tuberculosis* growth was significantly enhanced in Siglec-14 siRNA–transfected MDMs compared with control siRNA–transfected MDMs (Figure 4A, *P* = 0.039). RPS26, RGCC1, and ANXA1 siRNAs did not affect *M. tuberculosis* growth (Figure 4A).

### Siglec-14 reduces M. tuberculosis growth in MDMs through antimicrobial peptide production.

Ingenuity pathway analysis demonstrated the possible functional role of Siglec-14 in immunity and its downstream targets (Supplemental Figure 7). CD14^+^Siglec-14^+^ cells exhibited a differential phenotype and highly expressed *CCR5*, *CXCR1*, *CD200*, *PTPRC*, *TNF-α*, and *CSF1* compared with CD14^+^Siglec-14^–^ (Supplemental Figure 8). Next, we determined the mechanisms by which Siglec-14^+^ cells reduce *M. tuberculosis* growth in macrophages. MDMs of healthy individuals were transfected with control or Siglec-14 siRNAs. siRNA-transfected MDMs were infected with *M. tuberculosis* H37Rv at an MOI of 2.5. As shown in Figure 4, B–D, and Supplemental Figure 9, at 72 hours postinfection, control or Siglec-14 siRNA–treated and *M. tuberculosis*–infected MDMs did not differ in apoptosis, cytokine and chemokine production, autophagy, or opsonization of *M. tuberculosis* in the presence or absence of plasma from nonconverters and converters. As shown in Figure 4E, in the above-cultured cells, Siglec-14 siRNA significantly inhibited antimicrobial peptide gene expression by *M. tuberculosis*–infected MDMs compared with control siRNA–transfected, *M. tuberculosis*–infected MDMs. We also sorted CD14^+^Siglec-14^–^ and CD14^+^Siglec-14^+^ monocytes from freshly isolated PBMCs by flow cytometry and infected them with *M. tuberculosis* H37Rv at an MOI of 2.5. At 5 days postinfection, CD14^+^Siglec-14^+^ cells significantly restricted *M. tuberculosis* growth (*P* = 0.0003) and produced a higher level of antimicrobial peptides than CD14^+^ Siglec-14^–^ cells (Figure 4, F and G, and Supplemental Figure 10, *P* < 0.05).

### Nonconverters exhibit differential plasma metabolomic signatures.

**Figure 5:**
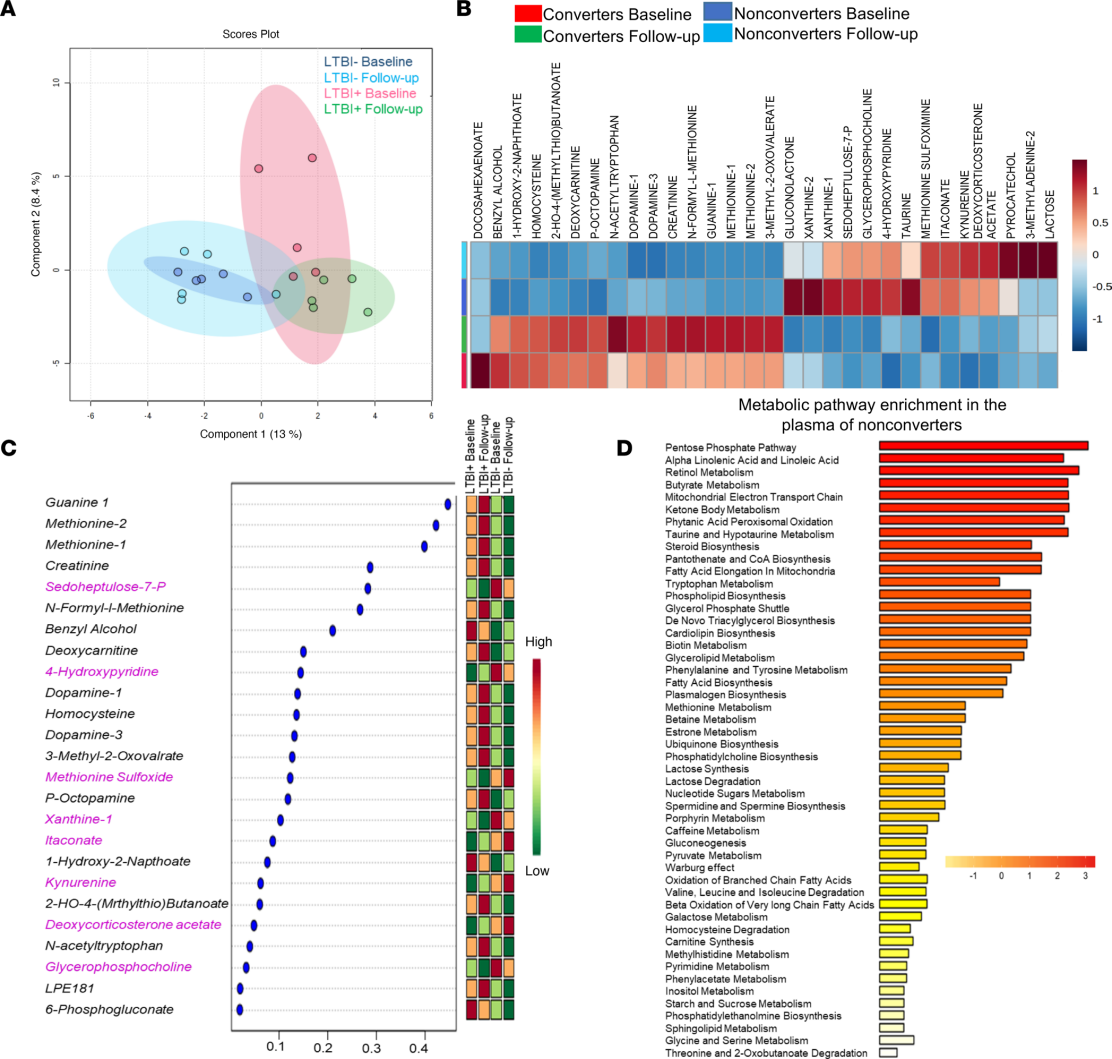
Nonconverters exhibit differential plasma metabolomic signatures. Lyophilized plasma from nonconverters (*n* = 5) and converters (*n* = 5) at baseline (0, at enrollment) and follow-up (24 months) was analyzed using LC-MS. (**A**) A representative score plot of the partial least squares discriminant analysis (PLS-DA) was generated using MetaboAnalyst. PLS-DA models were validated using R2 and Q2 based on leave-one-out cross-validation; the 4-component model was selected as the optimized model with R2 = 0.95 and Q2 = 058. The significance of the model was demonstrated by a permutation test with 100 testing iterations using a separation distance of *P* < 0.01 (95% confidence interval). Blue: nonconverter baseline, light blue: nonconverter follow-up, red: converter baseline, green: converter follow-up. (**B**) An FDR-corrected heatmap of selected metabolites is shown, *q* = 0.05. (**C**) Representation of 25 metabolites with variable importance of projection (VIP) scores based on PLS-DA and considered significant. On the extreme right, red indicates high levels, and green indicates low levels of metabolites in the respective groups. (**D**) Quantitative metabolite set enrichment overview using metabolite set enrichment analysis, with the fold change showing metabolic pathways of 25 metabolites selected based on VIP scores (FDR-corrected *q* = 0.05).

Metabolic changes can regulate the gene expression profile and immune responses to infection (31). Based on the above findings, we sought to determine whether nonconverters have differential plasma metabolomic profiles compared with converters. Plasma from converters (*n* = 5) and nonconverters (*n* = 5) at baseline (0 months) and during follow-up (24 months) was analyzed by liquid chromatography–mass spectrometry (LC-MS). Using a sparse partial least squares discriminant analysis algorithm that can effectively reduce the number of variables (metabolites) in high-dimensional metabolomics data to produce robust and easy-to-interpret models, we found that most of the data were within the 95% confidence region. Metabolites in the 2 groups (baseline and follow-up) were closely related to each other (Figure 5A). There was a minor overlap between the plasma samples from converters at baseline and follow-up and plasma from nonconverters at baseline and follow-up (Figure 5A). In contrast, a high level of segregation in the metabolic profile was noted between the converters and nonconverters at baseline and during follow-up applying ANOVA at a significance threshold of *P* < 0.05 (Figure 5B). Among more than 350 metabolites, 47 metabolites were identified, and a graphical representation of the individual metabolite levels in converters and nonconverters at baseline and during follow-up is provided as a heatmap (Figure 5B), showing the relative concentration of the metabolites (increase and decrease) across different groups. Among the segregated groups, using the variable importance in projection score, we identified 8 metabolites significantly (*P* < 0.05) higher at baseline and follow-up in the plasma of converters than nonconverter plasma (Figure 5C). Quantitative metabolite enrichment analysis was performed, and the metabolic pathways enriched in nonconverters compared with converters are represented (Figure 5D). All significant metabolites were mapped to the biological pathways in the Kyoto Encyclopedia of Genes and Genomes database, ultimately identifying 30 pathways (*P* < 0.05, ANOVA, Tukey’s *t* test). The results indicated that the pentose phosphate pathway and alpha-linolenic acid, linoleic acid, retinol, butyrate, and steroid biosynthesis pathways were active in nonconverters (Figure 5D). We selected the significantly (*P* < 0.05) higher metabolites, those that were elevated in the plasma samples of LTBI^–^ HHCs at baseline and follow-up. The metabolomic signature of exposed nonconverters and converters was different from that of unexposed healthy controls (Supplemental Figure 11).

### Deoxycorticosterone acetate enhances the expression of Siglec-14 by MDMs.

To determine the effect of the identified metabolites on Siglec-14 expression, we cultured CD14^+^ monocytes with γ-*M. tuberculosis* with or without the metabolites. The metabolites did not show any cytotoxic effects on the MDMs (Supplemental Figure 12). As shown in Figure 5A, among the 6 metabolites tested, only deoxycorticosterone acetate significantly enhanced Siglec-14 expression by MDM cultured in the presence of γ-*M. tuberculosis*. In adult humans, the level of deoxycorticosterone acetate ranges from 0.1 to 10 μM (32, 33), and with targeted metabolomics analysis in additional donors (*n* = 10), we verified that the level of deoxycorticosterone acetate was consistently higher in the plasma of nonconverters and converters at baseline and follow-up compared with converters (*P* = 0.0008, Supplemental Figure 11B). We also determined the concentration-dependent effect of deoxycorticosterone acetate on the expression of Siglec-14. We found that various doses, including physiological levels of deoxycorticosterone acetate, enhanced the expression of Siglec-14 (*P* < 0.05, *t* test, Supplemental Figure 13).

### Deoxycorticosterone acetate treatment promotes Siglec-14–dependent antibacterial activity in macrophages.

**Figure 6:**
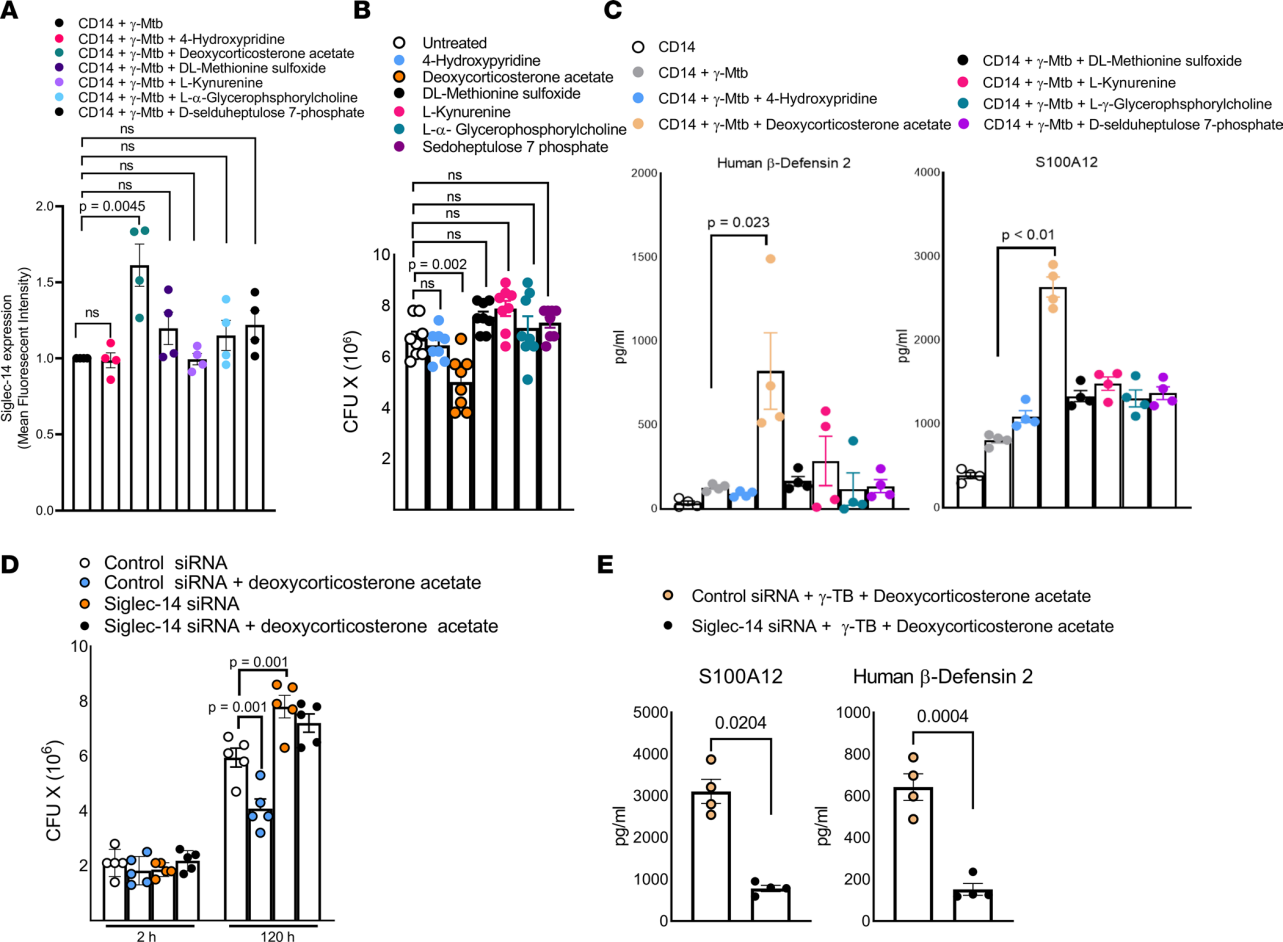
Deoxycorticosterone acetate treatment promotes Siglec-14–dependent antibacterial activity in macrophages. (**A**) Freshly prepared MDMs from healthy donors (*n* = 4) cultured in the presence or absence of γ-*M*. *tuberculosis* (10 μg/mL). Some γ-*M*. *tuberculosis*–cultured wells supplemented with metabolites were enriched in the plasma of nonconverters (4-hydroxypyridine, dl-methionine sulfoxide, l-kynurenine, l-α-glycerophosphocholine, d-sedoheptulose 7-phosphate, deoxycorticosterone acetate). After 72 hours, the expression (MFI) of Siglec-14 was determined by flow cytometry. (**B**) MDMs from healthy donors (*n* = 8) were infected at an MOI of 2.5. Some of the infected MDMs were cultured in the presence of the metabolites 4-hydroxypyridine, dl-methionine sulfoxide, l-kynurenine, l-α-glycerophosphocholine, d-sedoheptulose 7-phosphate, and deoxycorticosterone acetate (each 100 μM). Intracellular CFUs were determined at 5 days postinfection. *P* values were determined by unpaired 2-tailed *t* test. The mean ± SD is shown. (**C**) In the above panel, the supernatant was aspirated, and the level of HBD2 and S100A12 was determined by ELISA. *P* values were determined by unpaired 2-tailed *t* test. The mean ± SD is shown. (**D**) MDMs from healthy donors (*n* = 5) were isolated and transfected with siRNA targeting Siglec-14 or control siRNA and infected with *M*. *tuberculosis* H37Rv at an MOI of 2.5. In some *M*. *tuberculosis*–infected wells, deoxycorticosterone acetate (100 μM) was added. After 5 days, CFUs were counted. *P* values were determined by 1-way ANOVA with Tukey’s multiple comparisons test. Data are representative of 4 independent experiments. The mean ± SD is shown. (**E**) The concentrations of HBD2 and S100A12 were determined by ELISA. The *P* values were determined by unpaired *t* test. The mean ± SD is shown.

We determined the effect of the above metabolites on *M. tuberculosis* growth. MDMs were infected with *M. tuberculosis* H37Rv, and some of the infected wells were supplemented (as described in the Methods section) with the above 6 metabolites enriched in the plasma of nonconverters. As shown in Figure 6B, only deoxycorticosterone acetate significantly inhibited *M. tuberculosis* replication in MDMs, and none of the metabolites had a significant effect on cytokine production by *M. tuberculosis*–infected MDMs (Supplemental Figure 14). We also determined whether deoxycorticosterone acetate–mediated antimicrobial activity (Figure 6, B and C, and Supplemental Figure 15A) is dependent on Siglec-14 expression. MDMs from healthy individuals were transfected with siRNAs to Siglec-14 or control siRNA and infected with *M. tuberculosis* H37Rv at an MOI of 2.5. Some of the infected MDMs (Siglec-14 or control siRNA–transfected cells) were cultured with deoxycorticosterone acetate, as mentioned in the Methods. Five days postinfection, *M. tuberculosis* growth was significantly inhibited by deoxycorticosterone acetate and control siRNA–transfected MDMs but not in the Siglec-14 siRNA– and deoxycorticosterone acetate-treated MDMs (Figure 6D, *P* = 0.001). As shown in Figure 6C and Supplemental Figure 16, only deoxycorticosterone acetate treatment enhanced HBD2 and S100A12 antimicrobial peptide production by γ-*M. tuberculosis*–cultured MDMs, and this effect was inhibited by Siglec-14 siRNA (Figure 6E).

### Deoxycorticosterone acetate keeps MDMs in a glycolytic state.

**Figure 7:**
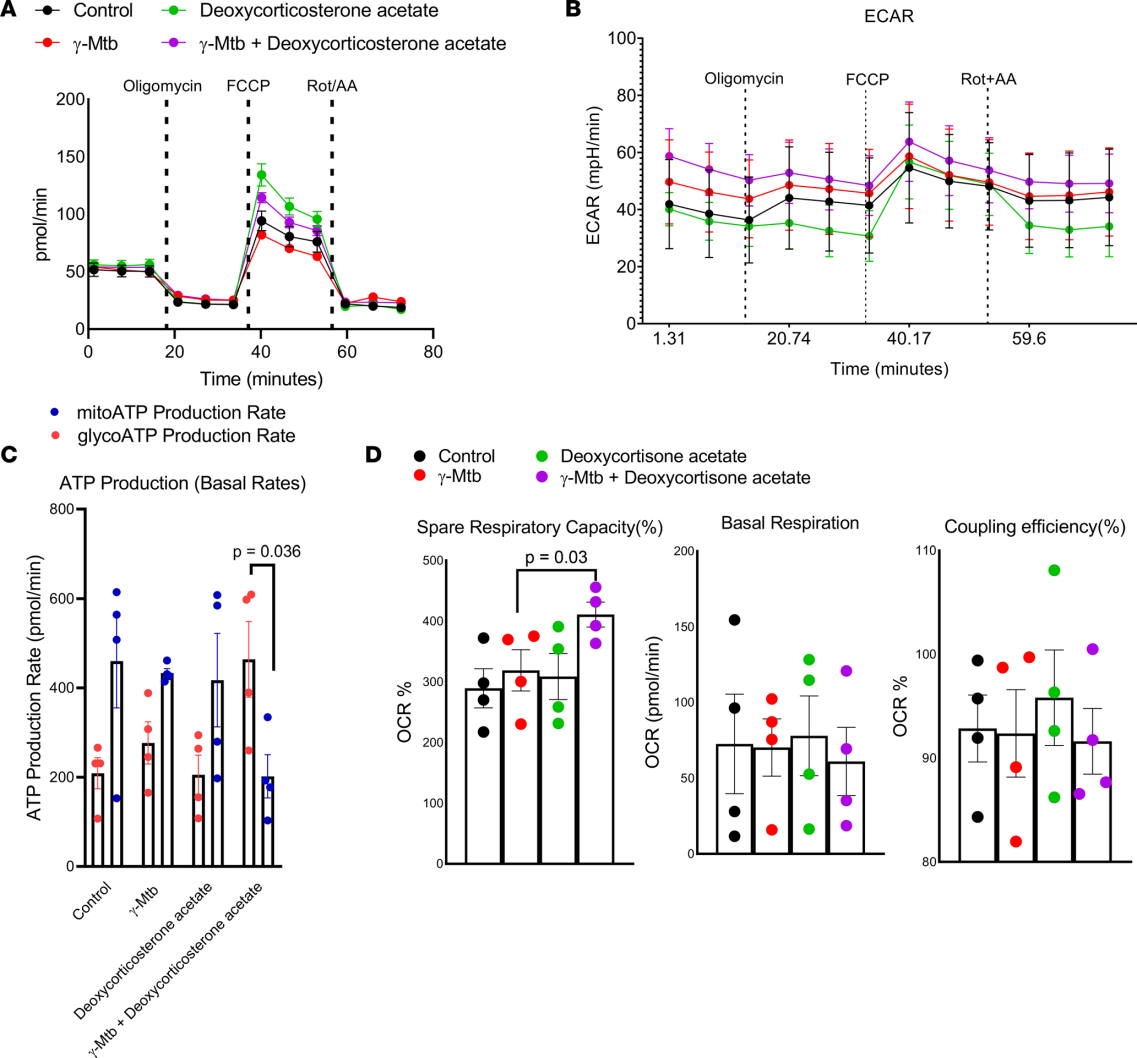
Deoxycorticosterone acetate keeps MDMs in a glycolytic state. MDMs were cultured in the presence or absence of γ-*M*. *tuberculosis* (10 μg/mL). Some γ-*M*. *tuberculosis*–cultured MDMs were cultured with or without deoxycorticosterone acetate (100 μM) and complete DMEM containing 10 mM glucose, 2 mM glutamine, and 2 mM sodium pyruvate as substrates. After 48 hours, (**A**) mitochondrial OCR and (**B**) ECAR were measured. (**C**) A bar graph showing the ratio of mitochondrial and glycolytic ATP is shown. The *P* values were derived using an unpaired 2-tailed independent *t* test. The mean values and SDs are shown. (**D**) A bar graph showing the SRC, basal respiration, and coupling efficiency. For all panels, the data are representative of 4 independent experiments. The *P* values were determined by 1-way ANOVA with Tukey’s multiple comparisons. The mean values and SDs are shown.

Deoxycorticosterone acetate treatment can elevate basic metabolic rates (34). We hypothesized that the deoxycorticosterone acetate–mediated antibacterial activity of MDMs could be due to changes of basic metabolic rates. We determined whether deoxycorticosterone acetate affects the metabolic state of γ-*M. tuberculosis*–cultured MDMs. We performed a metabolic flux assay (as mentioned in the Methods section) to detect changes in the mitochondrial oxygen consumption rate (OCR) and rate of extracellular acidification (ECAR) as measures of oxidative phosphorylation and glycolysis, respectively. Among all 6 tested metabolites, deoxycorticosterone acetate significantly increased the glyco-ATP production and spare respiratory capacity (SRC) (Figure 7, C and D). Simultaneously, deoxycorticosterone acetate enhanced the basal ECAR in γ-*M. tuberculosis*–cultured MDMs (Figure 7, A–D, and Supplemental Figure 17). These findings suggest that deoxycorticosterone acetate keeps γ-*M. tuberculosis*–cultured MDMs in the active glycolytic state in nonprogressors during *M. tuberculosis* exposure. Glycolysis converts glucose to pyruvate, which is subsequently oxidized by mitochondrial enzymes to generate bulk ATP (35). We also found a similar effect of deoxycorticosterone acetate on the γ-*M. tuberculosis*–cultured MDMs from converters in reducing oxidative phosphorylation; however, it did not affect the glycolytic flux (Supplemental Figure 18). In accordance with this, our transcriptomic data (metabolomic transcript analysis) demonstrated that the mitochondrial electron transport genes *ND2*, *ND3*, and *ND4*, which encode the enzyme NADH dehydrogenase (ubiquinone), and *COX1* and *COX2*, which encode cytochrome *c* oxidase, were elevated in the cells from nonconverters at baseline and follow-up compared with those from converters and active TB progressors (Supplemental Figure 19).

### 4-Hydroxypyridine enhances the expansion of memory-like NK cells.

**Figure 8:**
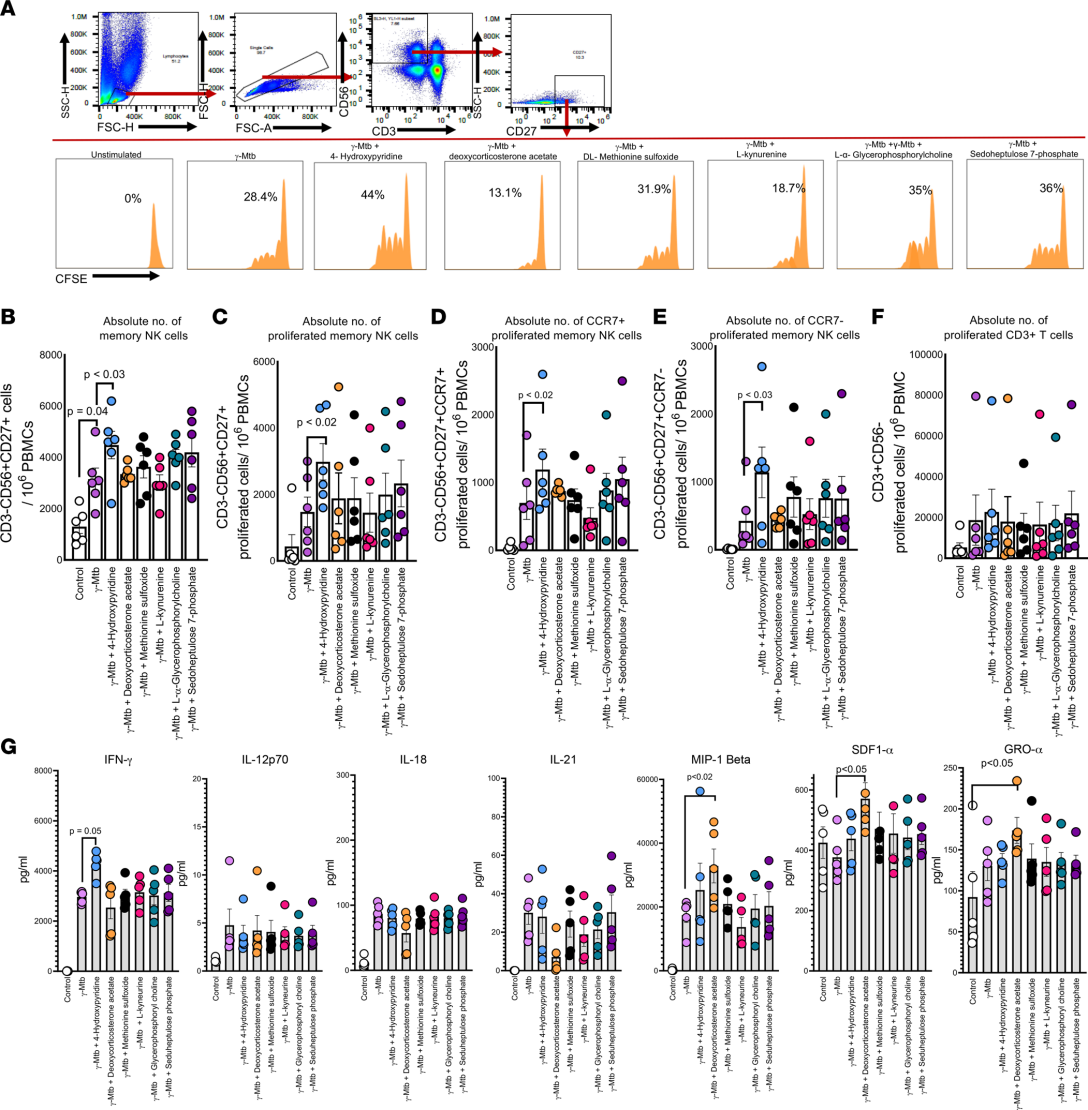
Metabolites enhance the expansion of memory-like NK cells and cytokine production. PBMCs from LTBI^+^ donors (*n* = 6) were labeled with CFSE and cultured with or without γ-*M*. *tuberculosis*. Some wells were supplemented with metabolites that were highly enriched in the plasma of nonconverters (4-hydroxypyridine, deoxycorticosterone acetate, dl-methionine sulfoxide, l-kynurenine, l-α-glycerophosphocholine, and d-sedoheptulose 7-phosphate, each 100 μM). After 5 days, the proliferation of memory NK cells was measured by flow cytometry. (**A**) A representative flow cytometry plot is shown. NK cells were identified by sequential gating on the lymphocytic singlet population and then on CD3^−^CD56^+^ NK cells. The events within the gated CD3^–^CD56^+^ NK cells were analyzed for CFSE^+^ cells and plotted in dot plots. (**B**) The total absolute number of CFSE^+^CD3^–^CD56^+^CD27^+^ cells is shown. (**C**) Absolute number of proliferating CD3^–^CD56^+^CD27^+^ cells. (**D**) Absolute number of proliferating CD3^–^CD56^+^CD27^+^CCR7^+^ cells. (**E**) Absolute number of proliferating CD3^–^CD56^+^CD27^+^CCR7^–^ cells. (**F**) Absolute number of proliferating CD3^+^ T cells. (**G**) In the above panels, after 5 days, the supernatants were aspirated, and cytokine and chemokine production was measured by multiplex ELISA. The *P* values were derived using an unpaired 2-tailed independent *t* test. The mean values, SDs, and *P* values are shown.

In Figure 1B, we show that nonconverters had a higher number of CCR7^+^ memory-like NK cells in their PBMCs than converters at baseline. Metabolites can reprogram immune effector cells through epigenetic modifications and enhance their function and proliferation (36). We hypothesized that the differential metabolite signature in nonconverters may be responsible for the expansion and higher number of CCR7^+^ memory-like NK cells. We determined whether the above-identified metabolites had any effect on the expansion of memory-like NK cells. PBMCs from 6 individuals with LTBI were labeled with CFSE and cultured with or without γ-*M. tuberculosis*. Some wells were supplemented with the metabolites. After 5 days, the number of proliferating CD3^–^CD56^+^CD27^+^CCR7^+^ cells was measured by flow cytometry. Among the 6 metabolites tested, we found that 4-hydroxypyridine treatment significantly enhanced the expansion of memory-like NK cell populations in γ-*M. tuberculosis*–stimulated PBMCs (Figure 8, A–F, and Supplemental Figure 20). We also found that in the above culture supernatants, only 4-hydroxypyridine significantly enhanced IFN-γ production by γ-*M. tuberculosis*–stimulated PBMCs as measured by multiplex ELISA (Figure 8G and Supplemental Figure 21).

## Discussion

Limited information is available on the protective immune responses of HHCs of patients with TB who never develop LTBI or TB (5). A recent study demonstrated that LTBI^–^ individuals who remained LTBI^–^ after long-term follow-up have strong humoral and noncanonical cellular immunity to *M. tuberculosis*, suggesting that a deeper understanding of the immune responses of LTBI^–^ contacts of patients with TB using established cohorts is essential (8). In the current study, we developed an HHC cohort that included 452 LTBI^–^ HHCs who were followed up at regular 4-month intervals up to 2 years and determined the frequency of the various immune cell populations and transcriptome and metabolome signatures at baseline and during follow-up (24 months). We also determined the role of highly expressed genes and metabolites in immune regulation. Among various cell populations, CD14^+^ cells and CCR7-expressing CD3^–^CD56^+^CD27^+^ memory-like NK cells were significantly higher in the PBMCs of nonconverters than converters at baseline (Figure 1B). Among the 5 transcripts highly expressed by the PBMCs of nonconverters, Siglec-14 is involved in the inhibition of *M. tuberculosis* growth in MDMs by enhancing the production of antimicrobial peptides. In various plasma metabolites, the levels of 6 metabolites were significantly higher in nonconverters than in converters (*P* < 0.05). Among these metabolites, deoxycorticosterone acetate allowed *M. tuberculosis*–infected MDMs to remain in a metabolically active state and restrict *M. tuberculosis* growth by enhancing Siglec-14 expression. Another metabolite, 4-hydroxypyridine, increased the expansion of memory-like NK cells and enhanced IFN-γ production. Our findings demonstrate that metabolites in the plasma of nonconverters keep *M. tuberculosis*–infected macrophages in an active glycolytic state, enhance the genes responsible for restricting *M. tuberculosis* growth, and promote innate control of *M. tuberculosis* infection.

The frequency of nonconverters within heavily exposed environments differs across different population studies since the defining features of resistance to *M. tuberculosis* infection are not well established (5, 37–40). To avoid misclassification and determine the presence and prevalence of true nonconverters, we performed a longitudinal follow-up analysis. We repeated testing (every 4 months) to minimize false negatives by considering the duration and intensity of exposure to the index case as mentioned in previous studies (10–14). In our cohort during the initial screening, we found LTBI in 52% of the cohort population, suggesting a high prevalence and incidence rate of *M. tuberculosis* infection. Among 452 LTBI^–^ HHCs, 96 (21.2%) developed new LTBI during follow-up (converters), and 293 (64.8%) HHCs remained LTBI^–^ (nonconverters) over 2 years. All the recruited HHCs were healthy, with no signs or symptoms of clinical immune dysfunction. PBMCs from nonconverters did not produce IFN-γ in response to *M. tuberculosis* antigens (ESAT-6 and CFP-10) at all time points (Supplemental Data 1). More than 90% of the LTBI conversion occurred within 12 months of enrollment (Supplemental Data 1).

Immune responses influence the outcome of any disease, and an increased number of CD16^+^ and NK cells in the HHCs is associated with LTBI (41). In TB endemic countries, NK cell phenotypes and functional profiles are modified, and these immune phenotypes can be used for the diagnosis of TB (42–44). We found significantly higher numbers of CD16-expressing CD14^+^ cells and CCR7-expressing CD3^–^CD56^+^CD27^+^ memory-like NK cells in nonconverters than in converters at baseline (Figure 1B), suggesting that the immune cell phenotype can play a role in resistance to TB in nonconverters. The increased number of these cell populations did not correlate with the type of cytokines and chemokines the above cells usually produce in response to *M. tuberculosis* antigens (Figure 2). Among the various cytokines and chemokines measured in the culture supernatants of *M. tuberculosis* antigen-stimulated PBMCs of nonconverters and converters, only PBMCs from nonconverters produced higher levels of TNF-α at baseline. In accordance with our finding, Lenette L. Lu et al. also demonstrated the presence of a higher number of ESAT-6/CFP-10–specific, TNF-α–producing CD4^+^IL-2^+^CD40L/CD154^+^ T cells in IGRA nonconverters (8). However, we found that after 24 months, PBMCs from converters and nonconverters produced similar amounts of TNF-α (Figure 2) in response to *M. tuberculosis* antigens. This result suggests some role for trained immunity during early stages of infection in LTBI nonconverters.

Transcriptomics data have provided new insights into the gene signature associated with *M. tuberculosis* infection risk (45–49). Blood transcriptomic biomarkers can discriminate patients with TB from healthy individuals (50), and a 16-gene transcriptome signature identified in an adolescent cohort can predict the progression of active TB (51). To determine the mechanisms by which nonconverters resist *M. tuberculosis* infection, we performed transcriptomic analysis of PBMCs cultured with *M. tuberculosis* antigens. We identified 5 transcripts (*CES1*, *Siglec14*, *RPS26*, *ANXA1*, and *RGCC1*) that had the highest relative expression by nonconverters compared with converters at baseline and during follow-up. Among these transcripts, Siglec-14 was highly expressed by *M. tuberculosis*–infected MDMs and was involved in controlling *M. tuberculosis* growth by enhancing the production of antimicrobial peptides. Siglecs belong to the Ig-lectin family of proteins expressed on mammalian leukocytes, recognize sialic acid–bearing glycans, and modulate immune responses to pathogens (52–54). Siglec-14 is highly expressed on innate immune cells, especially monocytes and neutrophils, and shares similar ligand-binding domains with Siglec-5 but with opposing signaling functions (55, 56). Activation of Siglec-5 produces an antiinflammatory signal, while stimulation through Siglec-14 induces a proinflammatory signal (57). Human Siglec-14 was shown to interact with an immunoreceptor tyrosine-based activation motif–containing adaptor that triggers both activating and inhibitory signaling (58). The interaction of HSP70 with Siglec-14 induces regulatory proinflammatory signals (57). Siglec-14 also enhances NLRP3 inflammasome activation in macrophages (59). Due to the Siglec-14–null polymorphism, Siglec-14 is absent in some humans, a condition that is associated with *M. tuberculosis* growth in monocytes and susceptibility to TB (60). Our current findings further demonstrate that Siglec-14 plays an important role in restricting *M. tuberculosis* growth in human MDMs and plays an important role in innate protective immune responses in nonconverter HHCs of patients with TB.

Metabolic profiling and targeted metabolite functional studies in several diseases have identified the mechanistic role of metabolites (61–63). Metabolic profiling can discriminate between patients with TB and healthy individuals (64–66). A longitudinal multisite study of the HHCs of TB patients of an African cohort demonstrated that a prognostic metabolic signature can predict the progression of active TB disease in HHCs (67). Based on our above findings, we determined whether the metabolic signatures of nonconverters and converters differ at baseline and during follow-up using an untargeted metabolomics approach based on liquid chromatography coupled to high-resolution mass spectrometry. The principal component analysis of the plasma metabolites of nonconverters and converters was different from that of the nonexposed healthy controls (ANOVA, *P* < 0.05, Supplemental Figure 11A). There was a significantly higher level of 8 metabolites at baseline and follow-up in the plasma of nonconverters than converters. Among these top significantly enriched metabolites in the plasma of nonconverters, the endogenous neurosteroid deoxycorticosterone acetate enhanced the expression of Siglec-14 by γ-*M. tuberculosis*–cultured MDMs and induced antimicrobial peptide production in a Siglec-14–dependent manner to restrict *M. tuberculosis* growth (Figure 6). In humans, the level of deoxycorticosterone acetate ranges from 0.1 to 10 μM (32, 33). We selected the concentrations of deoxycorticosterone acetate based on their cytotoxicity (100% cells were viable until 72 hours) and physiological concentration in the plasma of healthy individuals. We found that various doses, including physiological levels of deoxycorticosterone acetate, enhanced the expression of Siglec-14 (Supplemental Figure 13). *M. tuberculosis* infection induces a quiescent energy phenotype in human MDMs and decelerates flux through glycolysis and the TCA cycle (68). Switching to increased glycolysis is critical to control *M. tuberculosis* replication in macrophages (68). Deoxycorticosterone acetate treatment increased glycolytic ATP production rates and SRC of γ-*M. tuberculosis*–cultured MDMs. Mapping genes in different metabolic pathways to RNA-sequencing data indicated that mitochondrial metabolism genes had a distinct expression pattern in the nonconverters compared with converters (Supplemental Figure 19). This analysis and our findings, shown in Figure 7, suggest that deoxycorticosterone acetate keeps *M. tuberculosis*–infected MDMs in a metabolically active state. Excess amounts of neurocorticosteroids, such as deoxycorticosterone acetate, can cause hypertension (69), although suboptimal doses can prevent cytolysis of mycobacteria-infected lung fibroblast cells and reduce bacterial growth in MDMs (70). These effects are mediated through glucocorticoid receptors (70). The nonconverters in the current cohort were healthy, suggesting that suboptimal doses of deoxycorticosterone acetate in plasma help fight against *M. tuberculosis* infection. Our findings identify the potentially novel role of deoxycorticosterone acetate in enhancing innate immune responses against *M. tuberculosis* infection. Another metabolite, 4-hydroxypyridine, significantly increased the expansion of memory-like NK cells and enhanced IFN-γ production (Figure 8). 4-Hydroxypyridine is known as a good source of carbon and energy (71), which suggests that this metabolite can alter the bioenergetic metabolism of NK cells to support expansion during *M. tuberculosis* infection. Metabolic adaptation of *M. tuberculosis* to the intracellular environment of host macrophages is critical for its pathogenicity (23). Phosphoenolpyruvate carboxykinase is essential for the growth of *M. tuberculosis* on fatty acids and catalyzes the TCA cycle–derived metabolites into gluconeogenic intermediates and its impact on *M. tuberculosis* growth and virulence (25). Increased metabolites deoxycorticosterone acetate and 4-hydroxypyridine in nonprogressors may regulate the metabolic adaptation of *M. tuberculosis* and prevent the early establishment of the infection.

Our findings demonstrate that higher levels of circulating metabolites can enhance innate immune responses through transcriptional programming to effectively fight *M. tuberculosis* infection in nonconverters. It is not known how the alterations in the above-identified metabolites occur. One possible mechanism may be global epigenetic modifications, such as DNA methylation and chromatin modification, which are directly influenced by the environment, can regulate metabolic changes (72–74), and play an important role in disease outcomes (75).

We used a well-characterized cohort of HHCs from patients with TB to identify *M. tuberculosis* infection resistance mechanisms in nonconverters. Our study had some limitations. Due to the cost limits of omics, such as transcriptomics and metabolomics, we used age-, sex-, and epidemiological risk–matched small representative sample sizes from each group at baseline and follow-up. We excluded all samples with comorbidities and confounding factors before performing various assays. A larger population size would have provided greater statistical power to our studies. However, this sample size allowed us to analyze various immune parameters in the same individuals at the same time point at baseline and follow-up. Despite these limitations, our cohort study described the role of metabolites in innate resistance to human *M. tuberculosis* infection.

In summary, using unbiased transcriptome and metabolomic approaches, we identified the immune mechanisms that mediate resistance to TB in a large population of HHCs who never developed LTBI or active TB. These findings lay the groundwork for the development of novel methods to stimulate macrophage- and memory-like NK cell–mediated immunity against TB that could contribute to the development of an effective vaccine to prevent TB.

## Methods

### Cohort description.

As part of the Regional Prospective Observational Research for Tuberculosis–India (RePORT-India) study, a well-defined cohort of HHCs of pulmonary index TB patients was established in Hyderabad, India. HHCs were identified as adults or children living with the patient any time within the 3 months prior to the diagnosis of TB. Of the 1,230 HHCs screened for eligibility, 990 HHCs belonging to 443 microbiologically confirmed pulmonary active TB patients were enrolled in the study after obtaining written informed consent. A schematic representation of the detailed study plan is shown in Figure 1A. Detailed demographic and clinical data were collected during enrollment. Blood was collected to perform various immune studies and laboratory investigations at baseline and every 4 months for 2 years. LTBI was determined by an in-house IGRA, and a repeat test was performed every 4 months to identify new LTBI converters and those who remained infection free throughout the study period.

### Index cases.

Newly diagnosed, sputum-positive pulmonary tuberculosis patients receiving anti-tuberculosis therapy (ATT) at the Directly Observed Treatment, Short-course centers under the revised national tuberculosis control program at TB Clinics in Hyderabad were recruited as index cases. As a part of our RePORT study, we have thus far enrolled patients from Designated Microscopic Centers covering a total population of 1 million. For the current proposed studies, we collected various samples, including PBMCs, from HHCs at different time points (0, 4, 8, 12, 16, 20, and 24 months).

### HHCs.

Household members residing in the same house as the index case for a minimum of 3 months prior to the date of the diagnosis of TB were identified. These individuals must share at least 5 meals per week with the index case and have no history of TB or ATT.

The median number of HHCs per index case was 3. From June 2014 through June 2019, we enrolled 990 HHCs (Figure 1) aged 6–73 years after obtaining their informed consent. The study was approved by the institutional ethics committees of Bhagwan Mahavir Medical Research Center (BMMRC) and Blue Peter Hospital Research Center-Lepra Society (BPHRC) in Hyderabad. The details of HHCs, including age; sex; history of bacillus Calmette-Guérin vaccination; and history of pulmonary TB, smoking, drinking, diabetes, and other immunosuppressive conditions, were collected and are shown in Supplemental Table 1.

### Scoring criteria for exposure.

To determine the exposure of HHCs to patients with TB, we considered various factors, such as the infectivity of the index case, proximity of the contact to the index case, number of active TB cases in the household, and duration of exposure to the index case, as mentioned previously (76).

### Initial evaluation of all blood donors and exclusion/inclusion criteria.

All participants were screened for diabetes and HIV infection (by ELISA). HHCs with HIV, diabetes, pregnancy, autoimmune diseases, or any other immunosuppressive conditions were excluded during screening. For individuals, an in-house IGRA test was performed as previously published (77). Those who were tested as LTBI^+^ were evaluated for TB by chest radiography and clinical evaluation. If they had no evidence of TB, they were considered to have LTBI. Our clinicians at BPHRC and BMMRC evaluated the health conditions of each individual before recruitment.

### Follow-up of the HHCs.

All HHCs were followed up every 0, 4, 8, 12, 16, 20, and 24 months after enrollment. At each visit, they were evaluated for LTBI and TB status as mentioned above, and 20 mL of blood was collected. All HHCs with signs and symptoms of possible TB were referred for clinical evaluation and treatment.

### Outcome determination.

The outcomes of the study were as follows: (i) completion of a 2-year follow-up, (ii) development of LTBI in HHCs, and/or (iii) withdrawal of participants from the study. LTBI converters were defined as those with new LTBI positivity during a retest after the one at baseline. These converters were tested at every follow-up to confirm that they sustained the IGRA conversion. Nonconverters were defined as those with repeated negative IGRA results in the in-house assay (77).

### TB exposure assessment.

TB exposure was assessed using a standardized questionnaire. As previously reported (76), in brief, the exposure score consists of 11 items, including the presence of cough, pulmonary TB, sputum positivity of the index case, if the index case was the HHC’s primary caregiver, number of meals shared with the index patient, sleep location of the HHCs, and whether the HHCs live in the same house as the index TB patient. The score for high exposure was defined as more than 6 for adults.

### Determination of LTBI in the HHCs.

LTBI in the HHCs was determined according to our previously published protocols (77). A total of 2 × 10^6^ human PBMCs were stimulated with and without 10 μg/mL CFP-10 and ESAT-6 antigens and incubated at 37°C for 96 hours. IFN-γ released by PBMCs was measured with a sandwich ELISA using a commercial human IFN-γ kit (eBioscience, Thermo Fisher Scientific) following the manufacturer’s instructions. The IFN-γ concentration was calculated using Microplate Manager (MPM) software version 6.1 (Bio-Rad). HHCs were categorized as either LTBI^–^ or LTBI^+^ depending on the IFN-γ value.

Detailed methods for the previous sections are provided in Supplemental Methods.

### Statistics.

Prism 8.2 (GraphPad Software) was used for the statistical analyses. Descriptive analyses were performed for all relevant variables prior to their inclusion in the analyses. Frequency counts and percentages were obtained for interval and ordinal categorical data. For continuous variables, the central tendency (mean, mode, and median) and dispersion (range, variance, standard deviation, SEM, and coefficient of variation) were calculated. Possible outliers and/or influential observations were identified, and their validity was double-checked using available records. Here, *P* values less than 0.05 were considered statistically significant. The results are expressed as the mean ± SD. For the data that were normally distributed, the comparisons between groups were assessed via 1-way ANOVA and repeated measures mixed-effects ANOVA followed by post hoc Tukey’s multiple comparisons test adjusting *P* values.

### Study approval.

From June 2014 through June 2019, we enrolled 990 HHCs (Figure 1) aged 6–73 years after obtaining written informed consent. The study was approved by the institutional ethics committees from BMMRC and BPHRC.

## Author contributions

RV, DT, and VLV devised the project and the main conceptual ideas, proofed the outline, and conceived and planned the experiments. DT, KPD, and VSKN planned and carried out the experiments, performed the analysis, and designed the figures. TM, PP, RKR, AV, VM, and AKB carried out the experiments. KPS, DT, KPD, and ID performed data analysis. MSA screened the patients and was involved in participant counseling, recruitment, and follow-up. BS and KPD edited the manuscript. DT, RV, and KPD drafted the manuscript. The order of coauthorship was based on the contribution of main conceptual idea, experimental designing, and drafting of manuscript.

## Supplementary Material

Supplemental data

Supplemental data set 1

Supplemental data set 2

## Figures and Tables

**Table 1:**
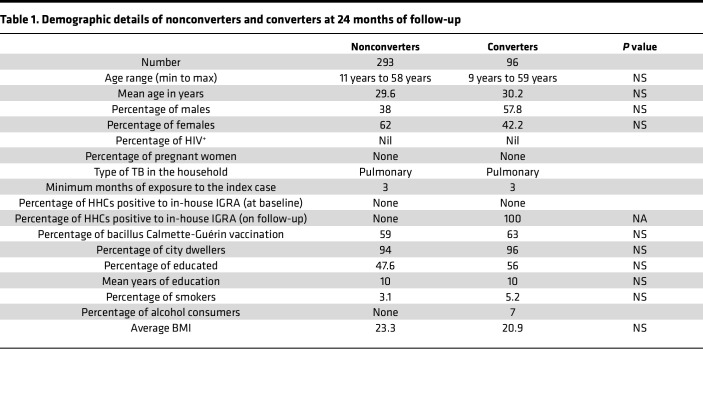
Demographic details of nonconverters and converters at 24 months of follow-up

## Version 1 11/22/2022

Electronic publication
