# Supplementary material for: Metabolites enhance innate resistance to human Mycobacterium tuberculosis infection
Source: JCI Insight. 2022 Nov 22;7(22):e152357. doi: 10.1172/jci.insight.152357 (PMC9746823; doi:10.1172/jci.insight.152357)
Supplement: Supplemental data [file jciinsight-7-152357-s020.pdf]

Supplemental Information

Supplemental Figures

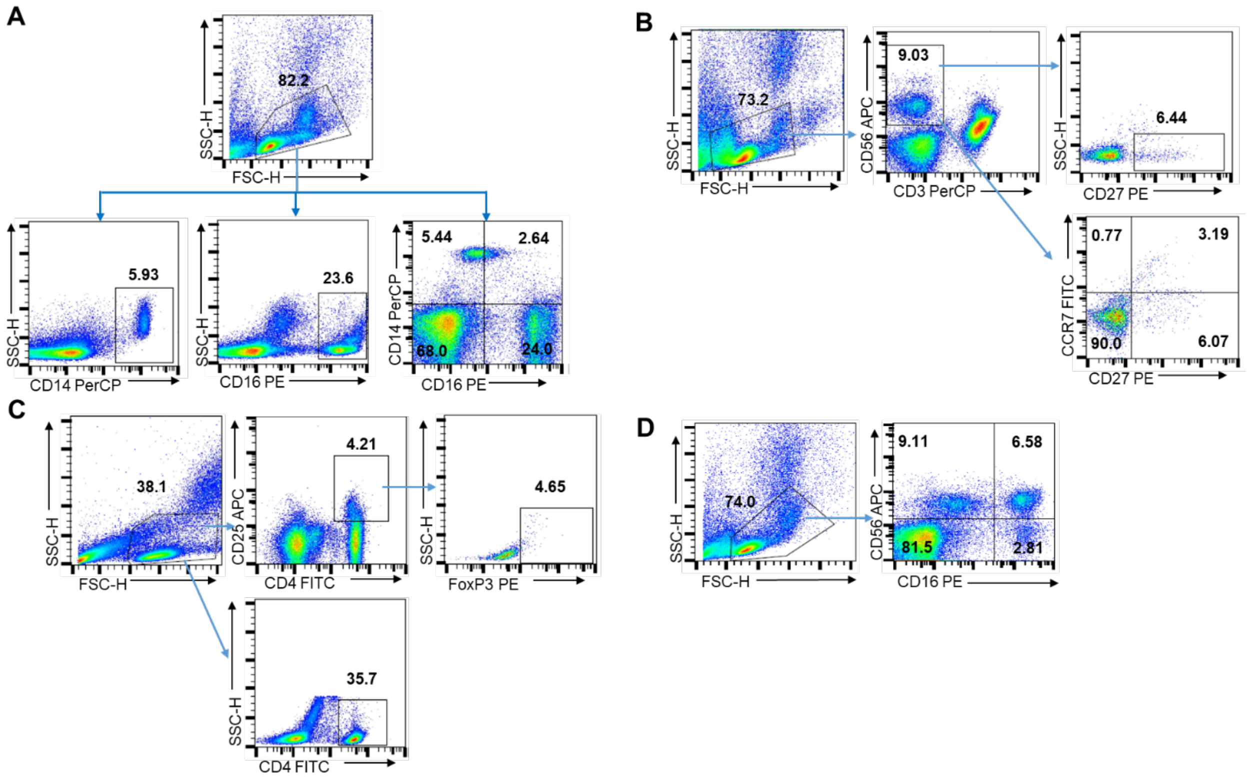

Figure S1 Representative flow cytometry figure showing the gating strategies. A representative flow cytometry figure for Figure 1B is shown. (A) CD14, CD16, CD14+CD16+ (B) CD3-CD56+CD27+ and CD3-CD56+CD27+CCR7+, (C) CD4+CD25+FoxP3+ and (D) CD16+CD56+ cells.

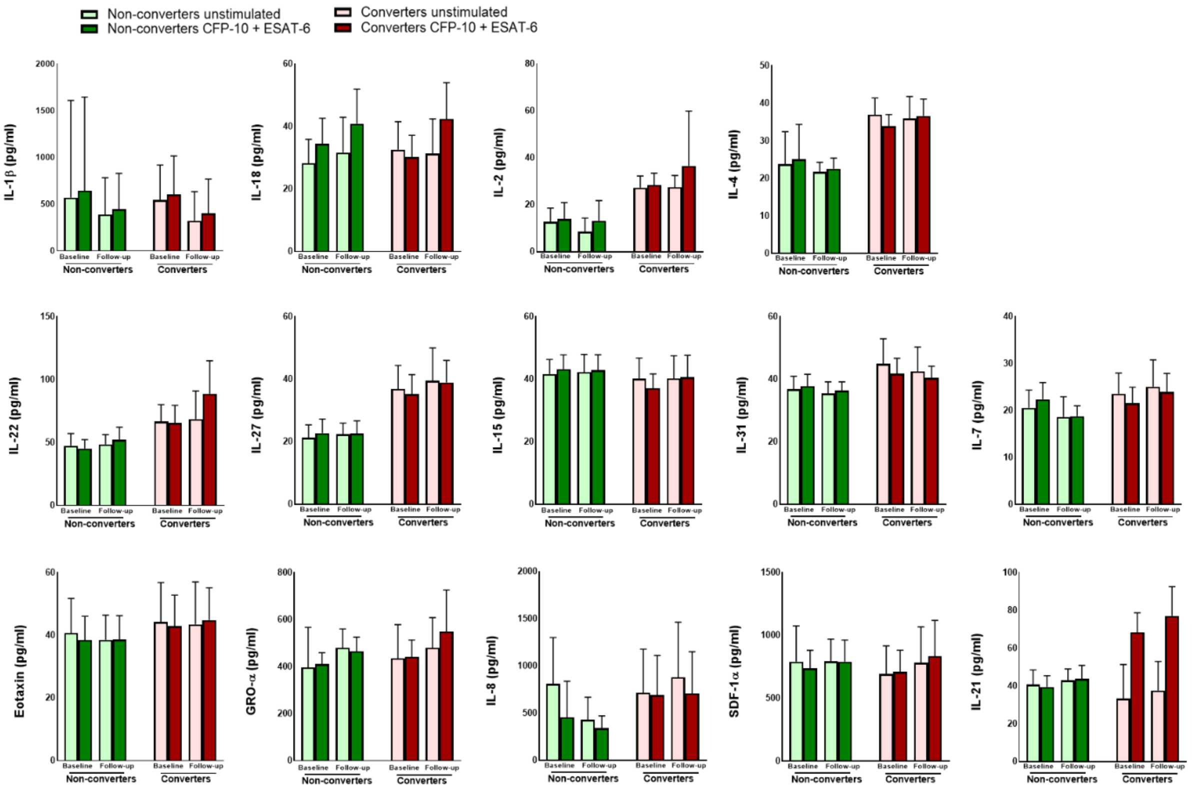

Figure S2 Cytokine and chemokine profiles of ESAT-6- and CFP-10-stimulated PBMCs. PBMCs from the nonconverters (n = 16) and converters (n = 16) at baseline and follow-up were isolated and cultured with or without ESAT6 and CFP10 (10 μg/ml each. After 96 h, the culture supernatants were collected, and the levels of the various chemokines and cytokines were measured using a multiplex enzyme-linked immunosorbent assay. The p values were determined by using repeated measures mixed-effects ANOVA with Tukey's multiple comparisons test. Mean values, SDs, and p values are shown.

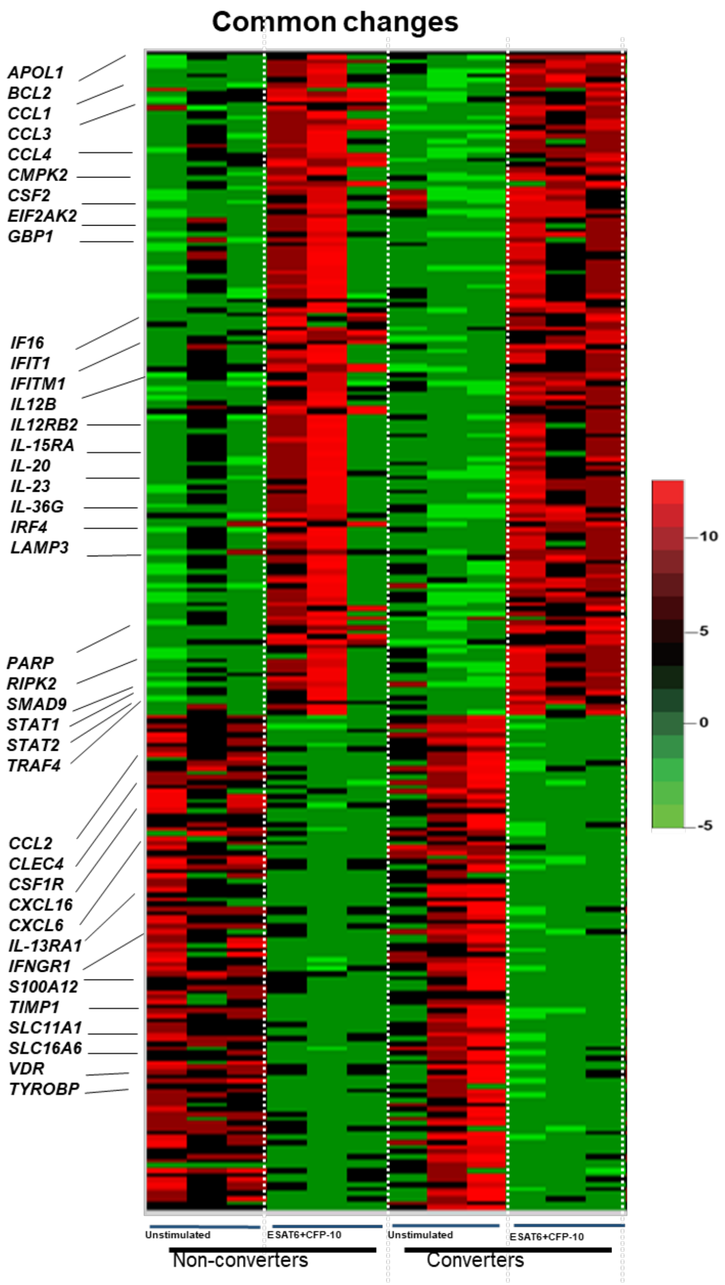

Figure S3 Common changes in the transcriptome of ESAT-6- and CFP-10-cultured PBMCs from nonconverters and converters. PBMCs from the nonconverters and converters at baseline were isolated and cultured with or without ESAT6 and CFP10 (10 µg/ml each). After 96 h, RNA was extracted. Common changes in the transcriptome were identified in the samples collected at baseline. Data are represented as a heatmap.

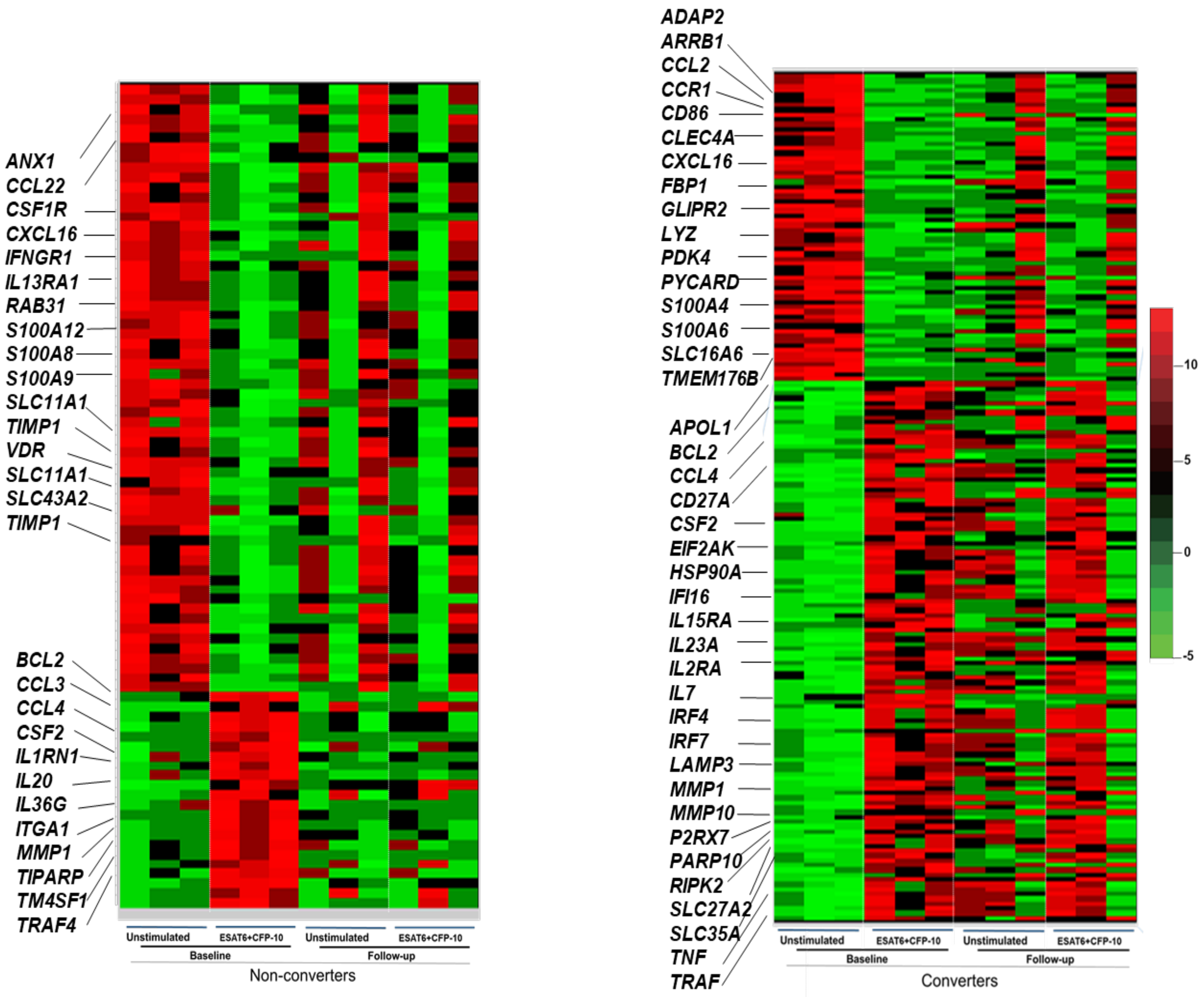

Figure S4 Unique changes in the transcriptome of ESAT-6- and CFP-10-cultured PBMCs from nonconverters and converters. PBMCs from the nonconverters and converters at baseline and follow-up were isolated and cultured with or without ESAT6 and CFP10 (10 µg/ml each). After 96 h, RNA was extracted. Unique changes in the transcriptome in the samples collected at baseline and the time points when LTBI- donors converted to LTBI+ and LTBI- donors remained LTBI- until 24 months were identified. Data are represented as a heatmap.

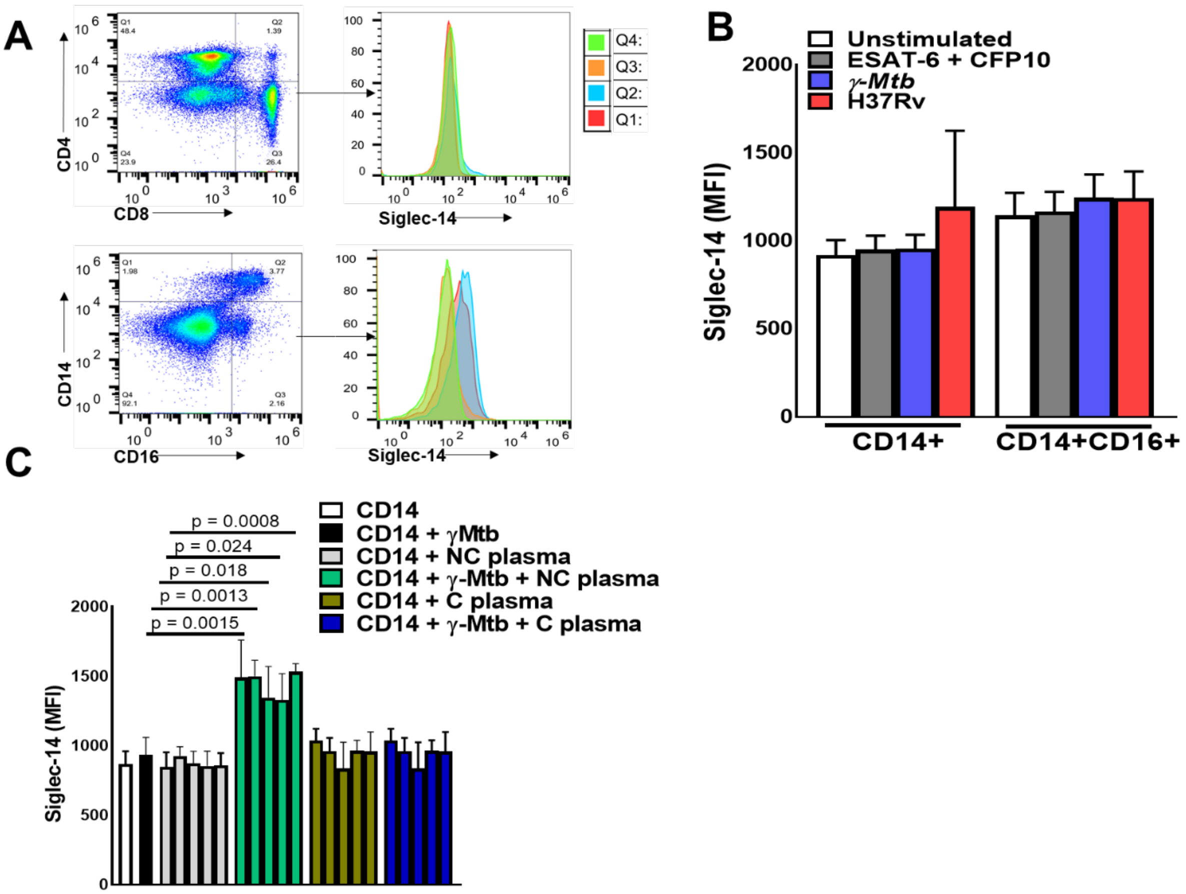

Figure S5 Siglec-14+-expressing immune cell populations. (A) PBMCs were obtained from LTB-nonexposed healthy donors (n = 3) and cultured in the presence or absence of γ-Mtb (10 μg/ml). After 96 h, the expression of Siglec-14 in various immune populations was determined by flow cytometry. A representative flow cytometry figure is shown. **(B)** CD14+ and CD14+CD16 cells were magnetically sorted and cultured in the presence or absence of ESAT-6+CFP10 (10 μg/ml) or γ-Mtb (10 μg/ml) or infected with MtbH37Rv at an MOI of 2.5. After 72 h, the expression of Siglec-14 was determined by flow cytometry. **(C)** Freshly isolated CD14+ cells were stimulated with γ-Mtb (10 μg/ml), and some wells were supplemented with inactivated plasma (2%) from nonconverters (n = 5) and converters (n = 5). After 72 h, the expression of Siglec-14 was determined by flow cytometry. Data are representative of 3 independent experiments. The p

values were determined by one-way ANOVA with Tukey's multiple comparisons test. The mean values, SDs, and p values are shown.

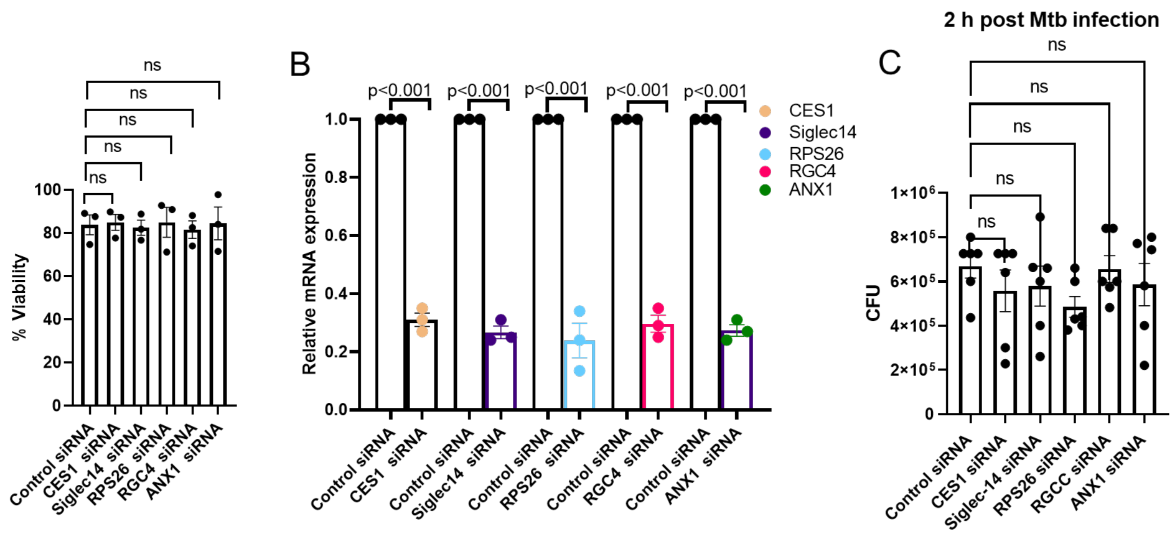

Figure S6. Effect of various siRNA treatments on the viability, knockdown efficiency and Mtb uptake of MDMs. Freshly isolated CD14+ cells from LTBI- healthy donors (n = 3) were differentiated into MDMs and transfected with siRNA targeting CES-1, Siglec-14, RPS-26, RGCC1, ANXA1, and control siRNA. **(A)** At 96 h post-transfection, the viability of MDMs was determined by the MTT assay. **(B)** At 96 h post-siRNA transfection, the knockdown efficiency of various siRNAs was determined by quantitative real-time PCR. **(C)** The siRNA-transfected MDMs (n = 6) were infected with H37Rv at an MOI of 2.5. After 2 h, the supernatant was aspirated, and the MDMs were lysed. The supernatant was centrifuged to pellet the bacteria, and the pellets were added to the cell lysates. The bacterial suspensions were ultrasonically dispersed, serially diluted, and plated in triplicate on 7H10 agar. The number of resultant colonies was counted after 3 weeks. The p values were derived using an unpaired two-tailed independent t test. The mean values and SDs are shown for the number of CFUs per well.

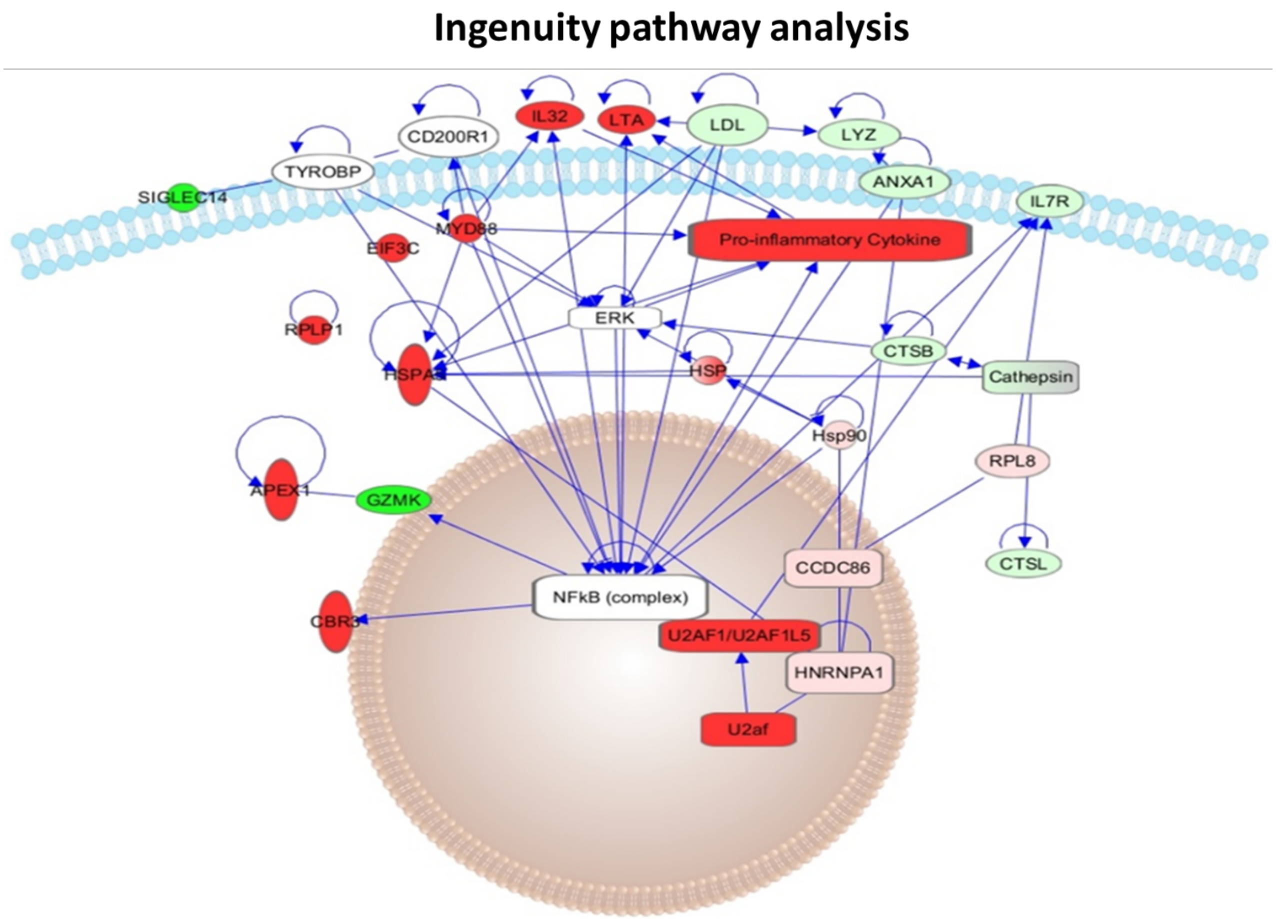

Figure S7 Ingenuity pathway analysis. The network of Siglec-14 depicted interactions between downstream genes and physiological functions. Red and green molecules indicate upregulated and downregulated genes in the nonconverters, respectively, relative to that in the converters. The figure legend displays molecules and functional symbol types and colors. The functional networks were generated through IPA (QIAGEN Inc., https://www.qiagenbio-informatics.com/products/ingenuity-pathway analysis).

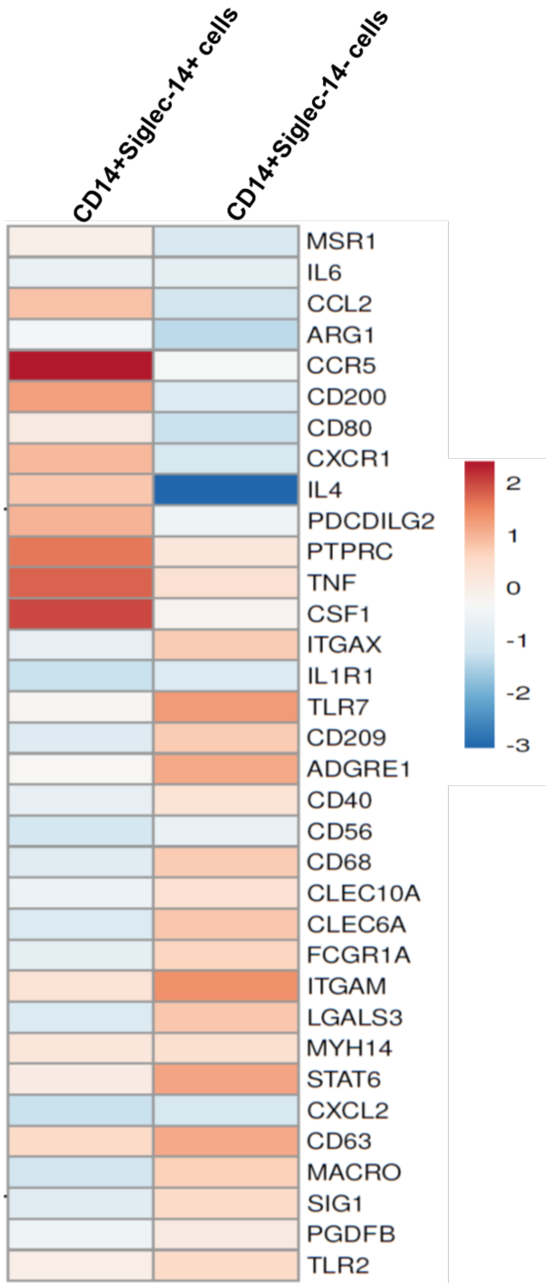

Figure S8. Gene signature of Siglec-14+ macrophages. CD14+Siglec-14+ and CD14+Siglec-14- cells were magnetically sorted from the PBMCs of healthy donors (n = 3). RNA was isolated from sorted CD14+Siglec-14+ and CD14+Siglec 14- cells, and quantitative real-time PCR was performed to determine the mRNA expression levels of various macrophage markers.

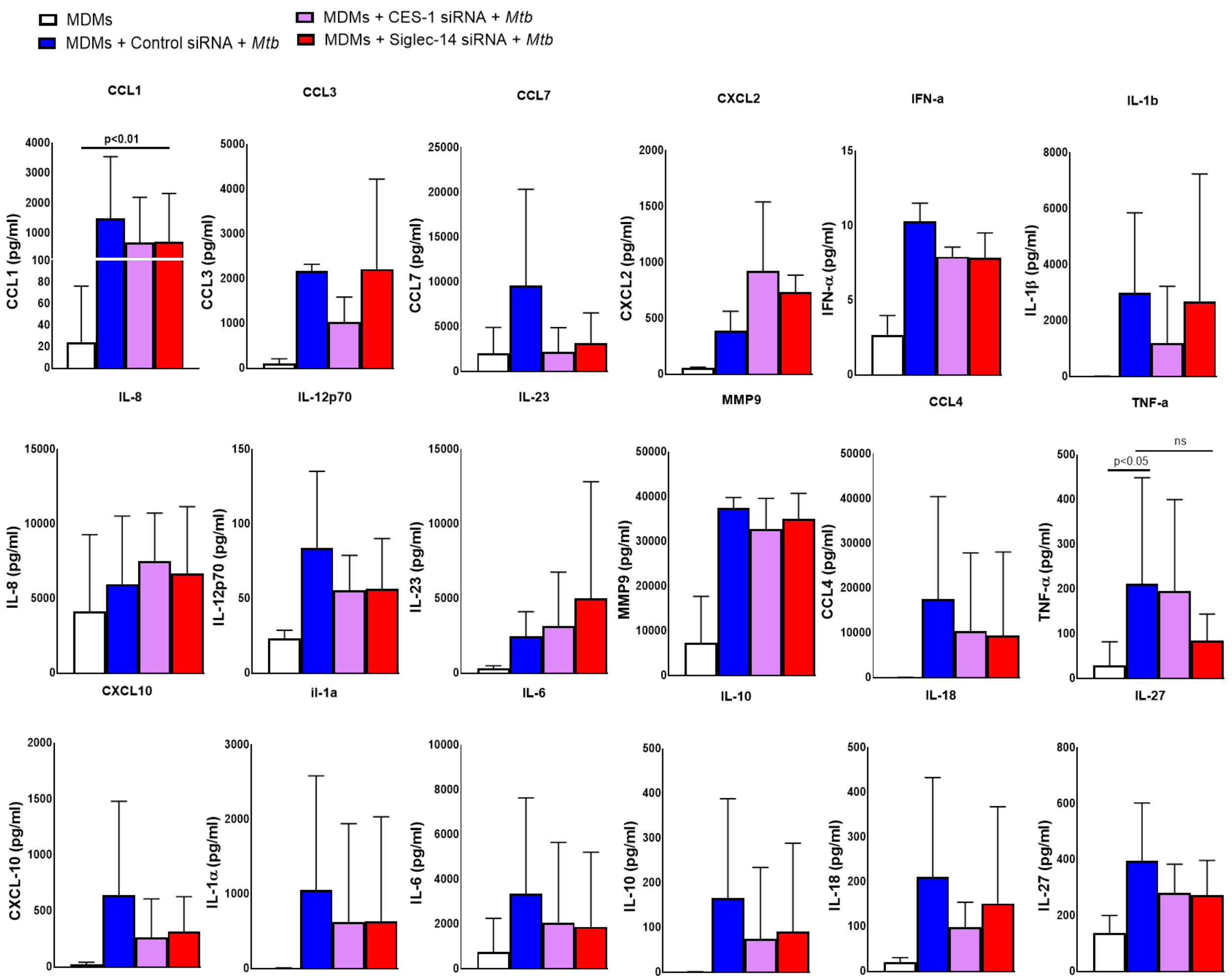

Figure S9 Siglec-14 siRNA does not affect cytokine and chemokine production by Mtb-infected MDMs. Control or Siglec-14 siRNA-transfected MDMs were infected with H37Rv at an MOI of 2.5 as described in the Methods. After 72 h, cytokine and chemokine levels in the culture supernatants were measured by multiplex ELISA. Mean values, SDs, and p values are shown.

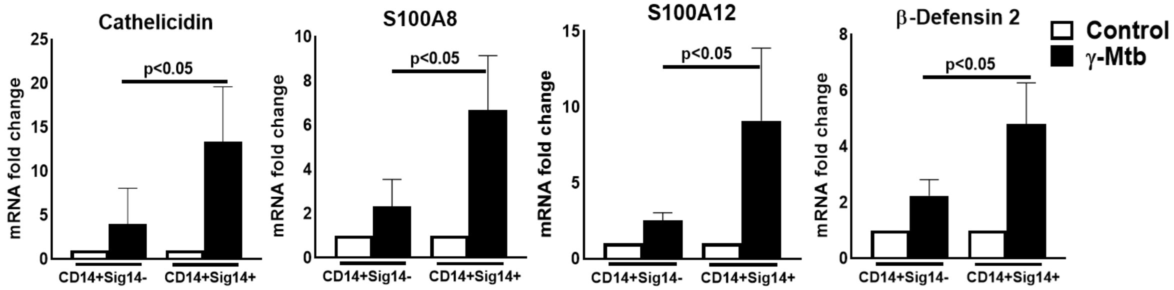

Figure S10 Siglec-14 cells exhibit an antimicrobial peptide gene signature. Freshly isolated PBMCs from LTBI- healthy donors (n = 5) were cultured in the presence or absence of γ-Mtb. After 72 h, CD14+Siglec-14+ and CD14+Siglec-14- cells were sorted through magnetic labeling, and RNA was isolated. Quantitative real-time PCR was performed to determine the mRNA expression level of antimicrobial peptides. The p values were determined by one-way ANOVA with Tukey's multiple comparisons test. Mean values, SDs, and p values are shown.

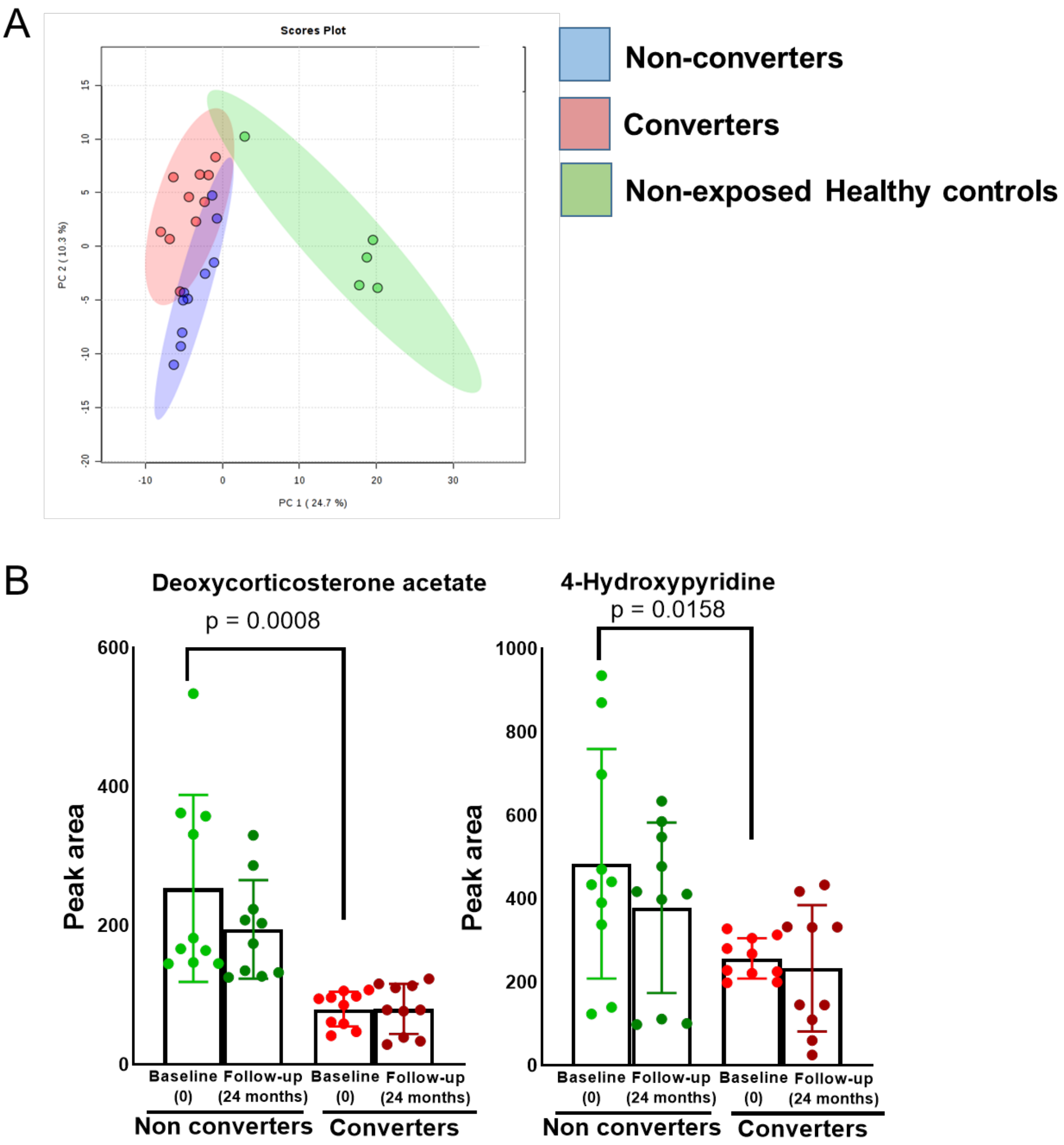

Figure S11 The plasma metabolomic signature of HHCs of TB patients is different from nonexposed healthy controls. Lyophilized plasma from the exposed households of TB patents (nonconverters: baseline, n = 5, follow-up, n = 5; converters: baseline, n = 5, follow-up, n = 5) and nonexposed healthy controls (n = 5) was analyzed using LC–MS. **(A)** A representative score plot of the partial least squares discriminant analysis (PLS-DA) was generated using MetaboAnalyst. PLS-DA models were validated using R2 and Q2 based on leave one out cross-validation

(LOOCV), and the four-component model was selected as the optimized model, with R2 = 0.95 and Q2 = 058. The significance of the model was demonstrated by a permutation test with 100 testing iterations using a separation distance of $p<0.01$. Blue: nonconverters, red: converter baseline, and green: nonexposed healthy controls. **(B)** The level of deoxycorticosterone acetate and 4-hydroxypyridine was quantified in the plasma sample of nonconverters (n = 10) and converters (n = 10) at baseline and follow-up using LC-MS.

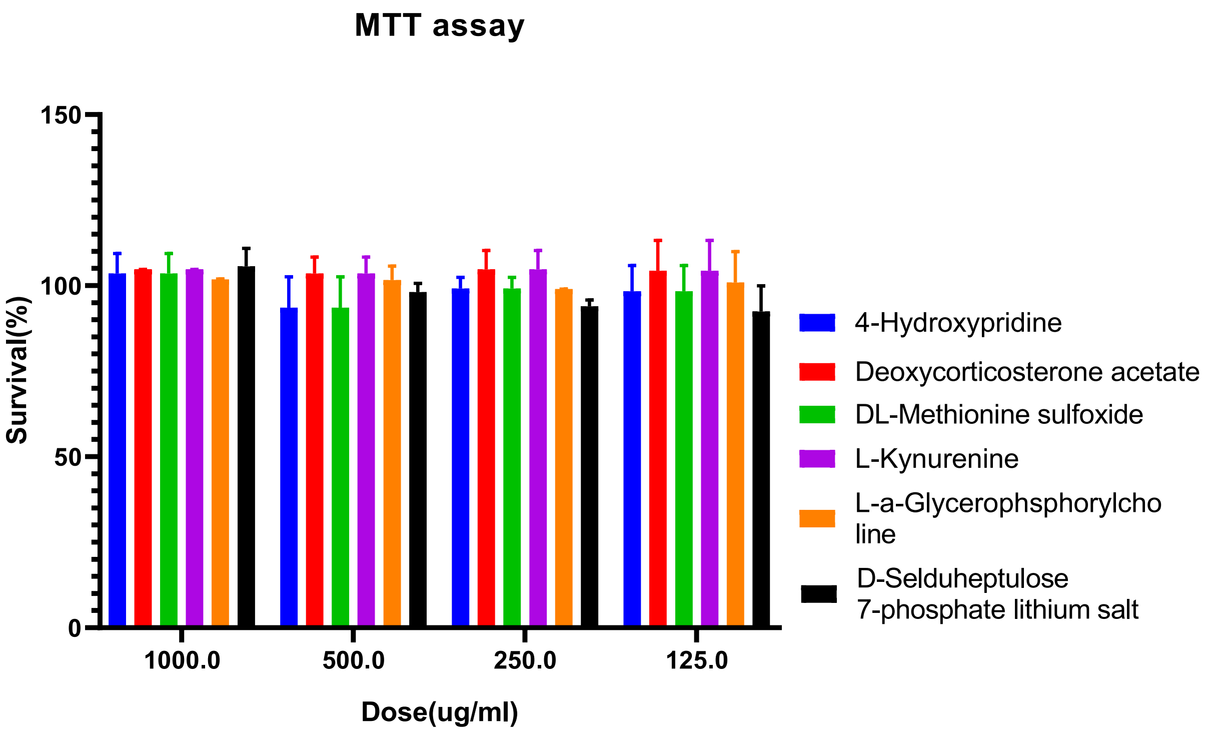

Figure S12 Cytotoxicity of the metabolites. Freshly isolated CD14+ cells were cultured in the presence of varying concentrations of metabolites (4-hydroxypyridine, DL-methionine sulfoxide, L-kynurenine, L-α-glycerophosphocholine, D-sedoheptulose 7-phosphate, deoxycorticosterone acetate). After 72 h, the cytotoxicity of metabolites relative to untreated CD14+ cells was determined by MTT assay. Data are representative of 3 independent experiments. The p values were determined by one-way ANOVA with Tukey's multiple comparisons test. Mean values, SDs, and p values are shown.

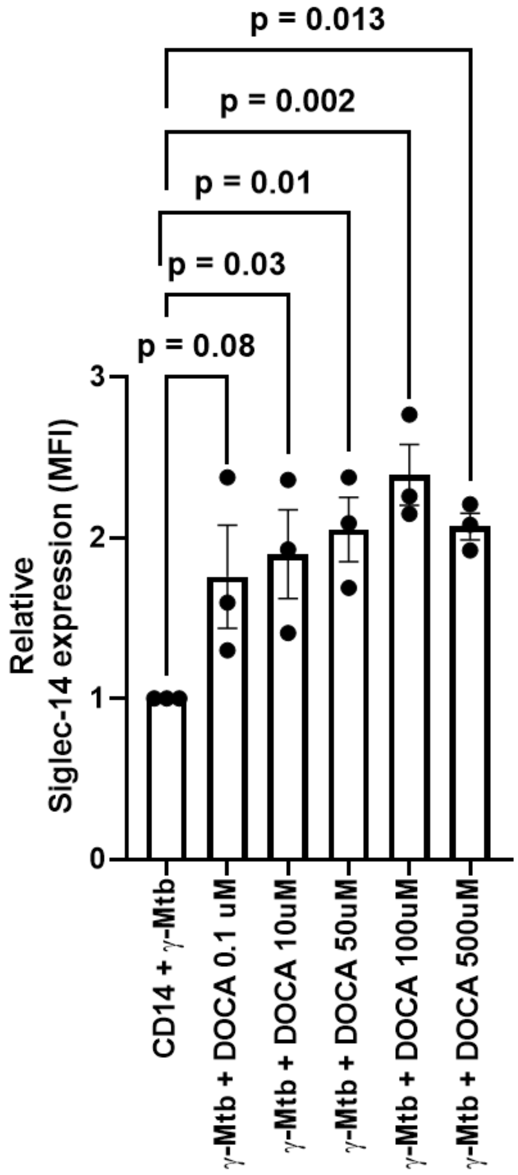

Figure S13 Deoxycorticosterone acetate treatment enhances Siglec-14 expression on macrophages. Freshly prepared MDMs from healthy donors (n = 3) cultured in the presence or absence of γ-Mtb (10 μg/ml). Some γ-Mtb cultured wells were supplemented with various concentrations of the metabolite deoxycorticosterone acetate. After 72 h, the expression (mean fluorescence intensity) of Siglec-14 was determined by flow cytometry. The mean ± SD is shown. The p values were determined by one-way ANOVA with Tukey's multiple comparisons test.

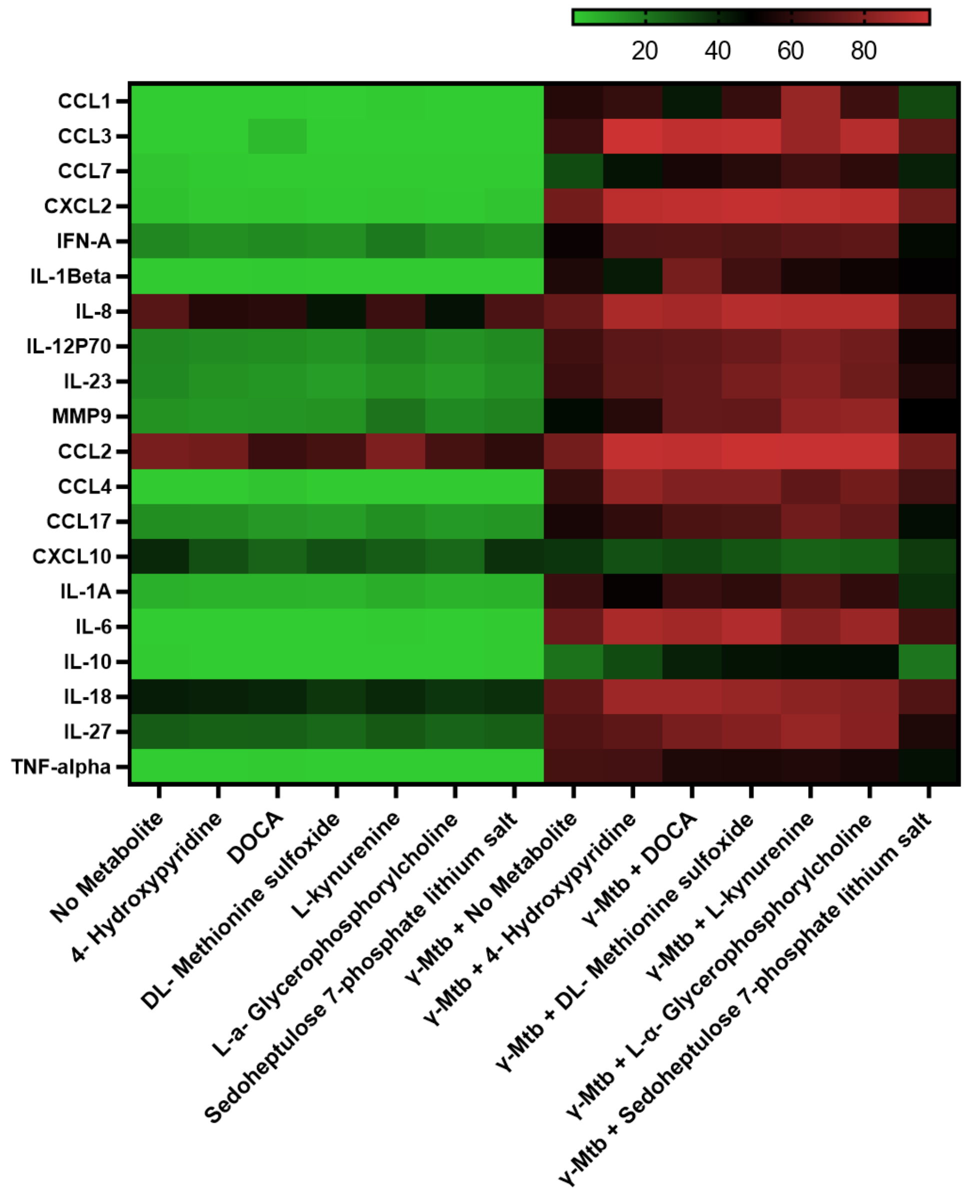

Figure S14 Cytokine and chemokine production by γ-Mtb-cultured MDMs in response to metabolites. Freshly isolated CD14+ cells were cultured with or without γ-Mtb. Some of the γ-Mtb cultured cells were supplemented with various metabolites (4-hydroxypyridine, DL-methionine sulfoxide, L-kynurenine, L-α-glycerophosphocholine, D-sedoheptulose 7-phosphate, deoxycorticosterone acetate). After 72 h, the culture supernatants were aspirated, and multiplex ELISA (20 plex) was performed to determine the levels of cytokines and chemokines.

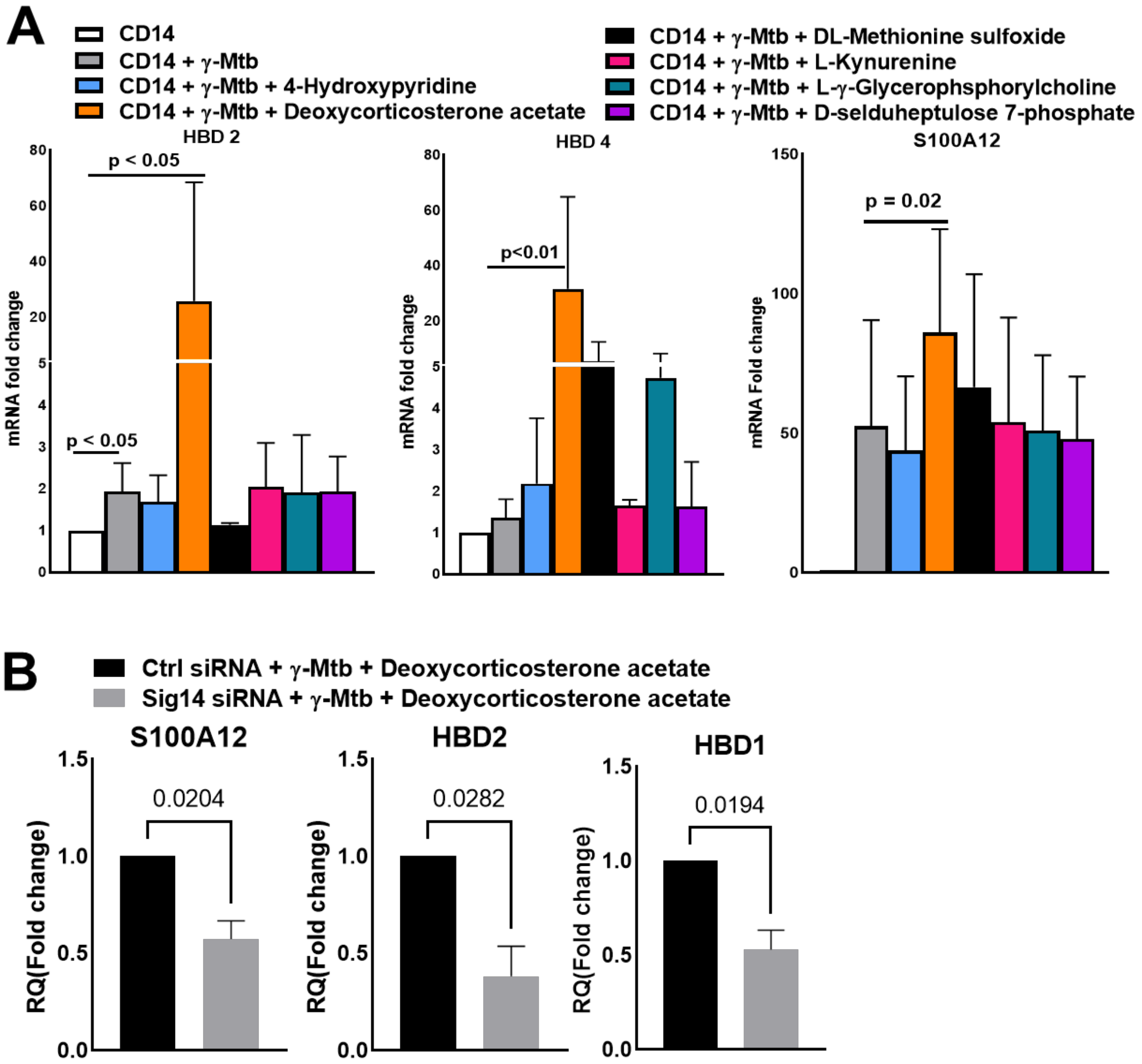

Figure S15 Deoxycorticosterone acetate treatment promotes Siglec-14-dependent antibacterial activity in macrophages. (A) MDMs were infected at an MOI of 2.5, as described in the Methods. Some of the infected MDMs were cultured in the presence of the metabolites 4-hydroxypyridine, DL-methionine sulfoxide, L-kynurenine, L-α-glycerophosphocholine, D-sedoheptulose 7-phosphate, and deoxycorticosterone acetate (each 100 μM). After 72 h, mRNA levels of HBD2, HBD4, and S100A12 were determined by real-time PCR. **(B)** MDMs from healthy donors (n = 4) were isolated and transfected with siRNA targeting Siglec-14 or control

siRNA and then infected with Mtb H37Rv at an MOI of 2.5. In some Mtb-infected wells, deoxycorticosterone acetate (100 μM) was added. After 5 days, the mRNA expression of HBD2, HBD4, and S100A12 was determined by real-time PCR. The p values were determined by one-way ANOVA with Tukey's multiple comparisons test. The mean ± SD is shown.

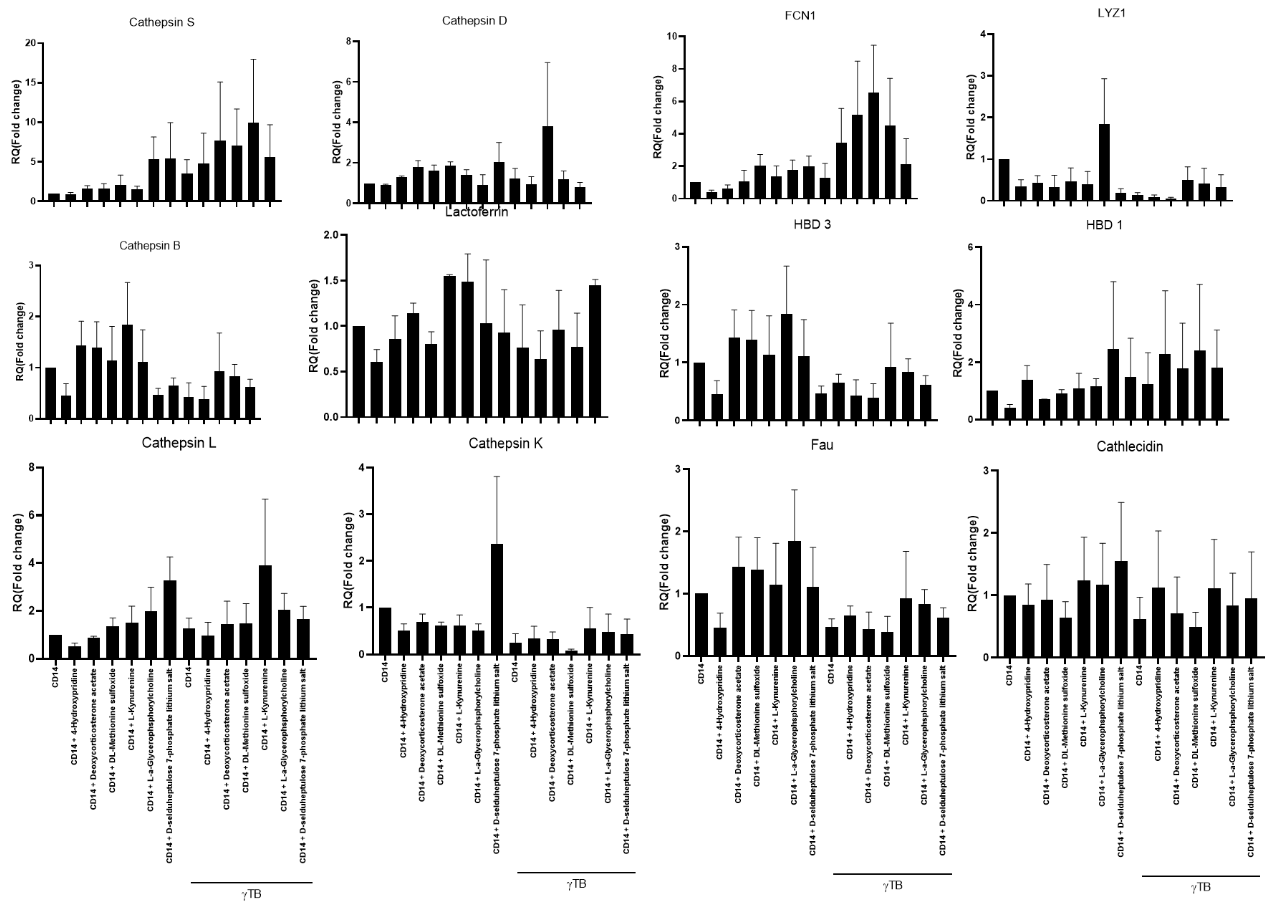

Figure S16 Antimicrobial peptide gene expression by γ-Mtb-cultured MDMs in response to metabolites. Freshly isolated CD14+ cells were cultured in the presence or absence of γ-Mtb, and some γ-Mtb-cultured MDMs were cultured with various metabolites (4-hydroxypyridine, DL-methionine sulfoxide, L-kynurenine, L-α-glycerophosphocholine, D-sedoheptulose 7-phosphate, deoxycorticosterone acetate). After 72 h, the expression of antimicrobial peptide mRNA was determined by real-time PCR. The p values were determined by one-way ANOVA with Tukey's multiple comparisons test. The mean ± SD is shown.

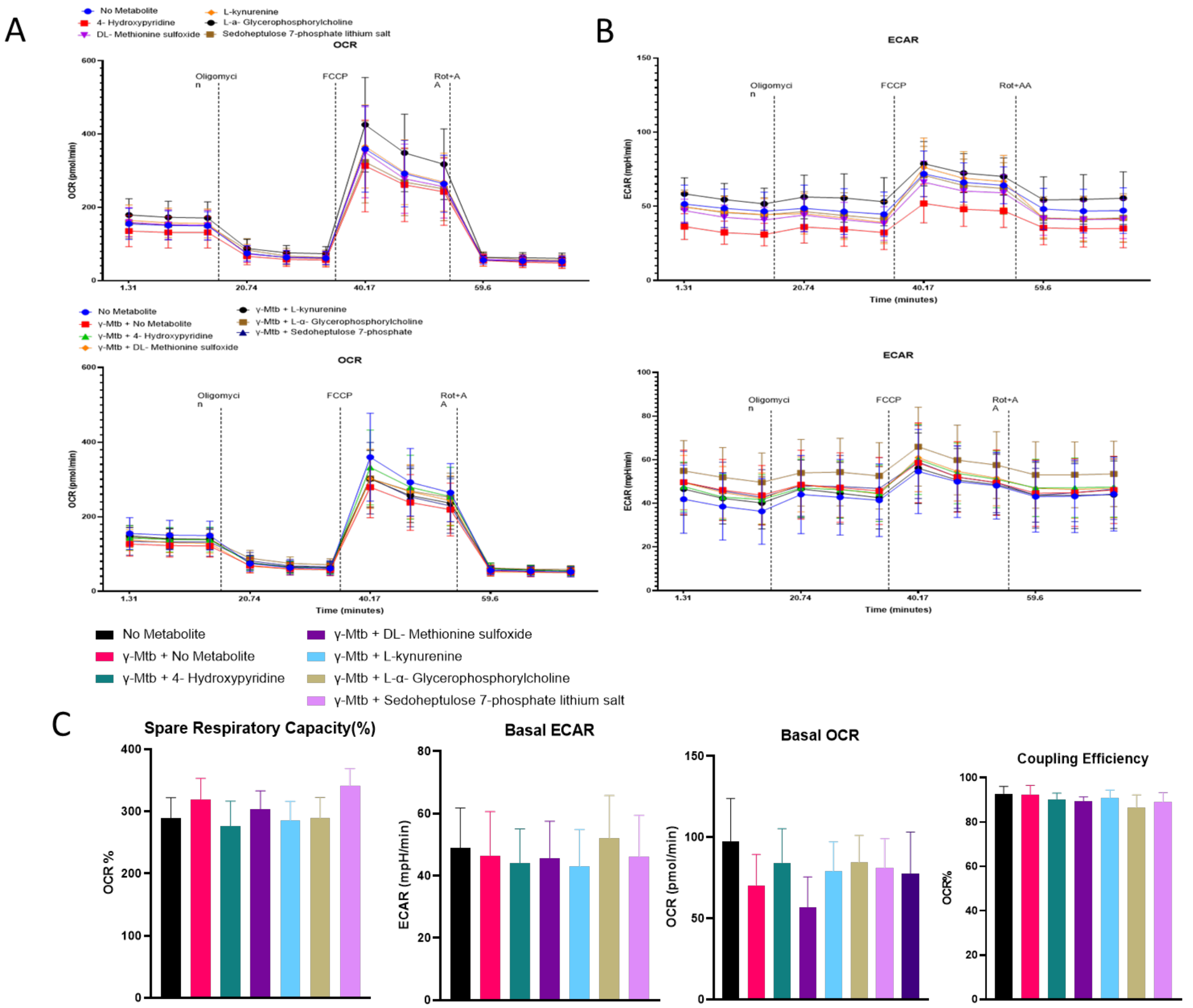

Figure S17 Metabolic activity of MDMs in the presence of metabolites. MDMs were cultured in the presence or absence of γ-Mtb (10 μg/ml). Some γ-Mtb-cultured MDMs were cultured with or without various metabolites (4-hydroxypyridine, DL-methionine sulfoxide, L-kynurenine, L-α-glycerophosphocholine, D-sedoheptulose 7-phosphate) and with complete DMEM containing 10 mM glucose, 2 mM glutamine, and 2 mM sodium pyruvate as substrates. After 48 h, **(A)** mitochondrial OCR and **(B)** ECAR were measured. **(C)** Bar graphs showing the spare respiratory capacity, basal ECAR, OCR and coupling efficiency are shown. For all panels, the data are

representative of 4 independent experiments. The mean ± SD is shown. The p values were determined by one-way ANOVA with Tukey's multiple comparisons test.

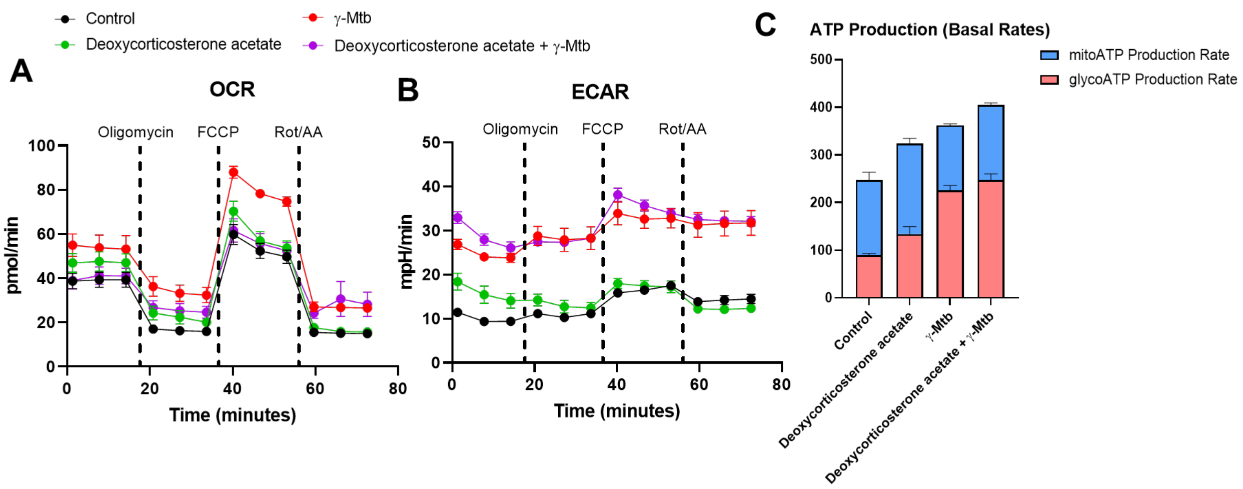

Figure S18 Metabolic activity of γ-Mtb-cultured MDMs from LTBI+ in the presence of metabolites. (A) MDMs from LTBI+ individuals (n = 4) were cultured in the presence or absence of γ-Mtb (10 μg/ml). Some γ-Mtb-cultured MDMs were cultured with or without various metabolite deoxycorticosterone acetate and with complete DMEM containing 10 mM glucose, 2 mM glutamine, and 2 mM sodium pyruvate as substrates. After 48 h, **(A)** mitochondrial OCR and **(B)** ECAR were measured. **(C)** A bar graph showing the ratio of mitochondrial and glycolytic ATP is shown. The mean ± SD is shown. The p values were determined by one-way ANOVA with Tukey's multiple comparisons test.

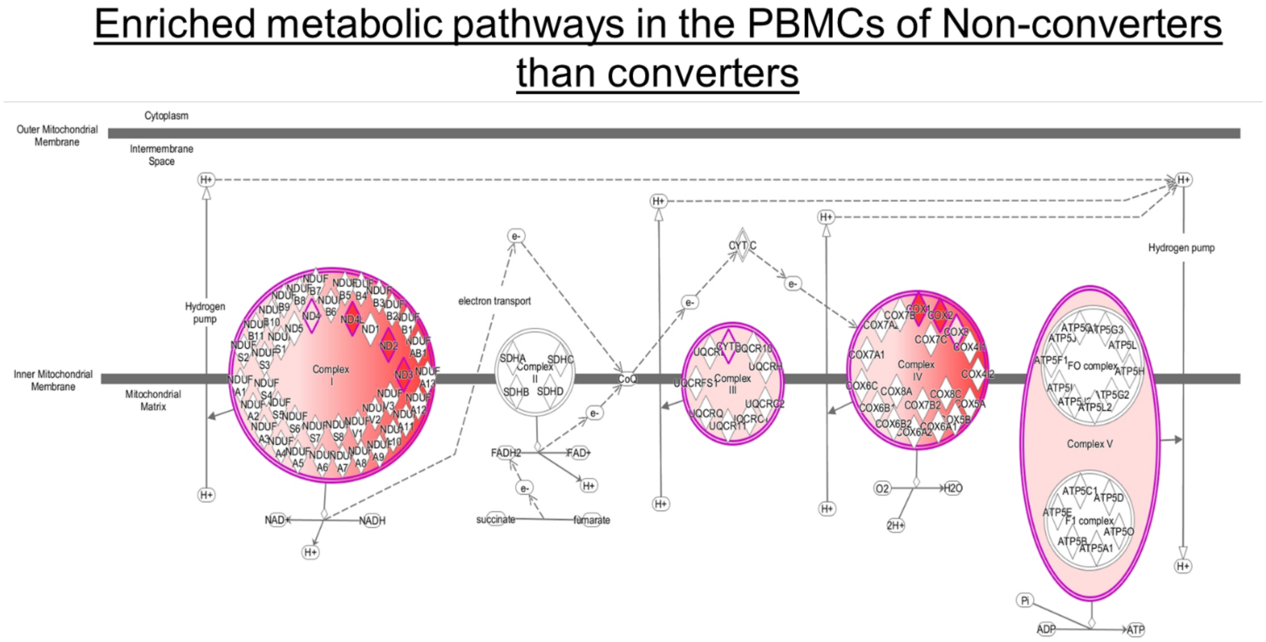

Figure S19 Metabolic pathways of ESAT-6- and CFP-10-cultured PBMCs of nonconverters and converters. PBMCs were isolated from age-matched, epidemiological risk-matched, healthy (no comorbid conditions and no immunosuppressive drugs) converters (n = 3) and nonconverters (n = 3) at baseline (during enrollment in the study, when all participants were healthy and LTBI-) and after 24 months. Freshly isolated PBMCs were cultured with or without ESAT-6 and CFP-10 (10 μg/ml each). After 96 h, total RNA was extracted, cDNA libraries were prepared, and whole-transcriptome sequencing was performed. A representative heatmap is shown. The pathway map (adapted from KEGG) shows that most of the upregulated (dark red) mitochondrial metabolism genes catalyze reactions in electron transport.

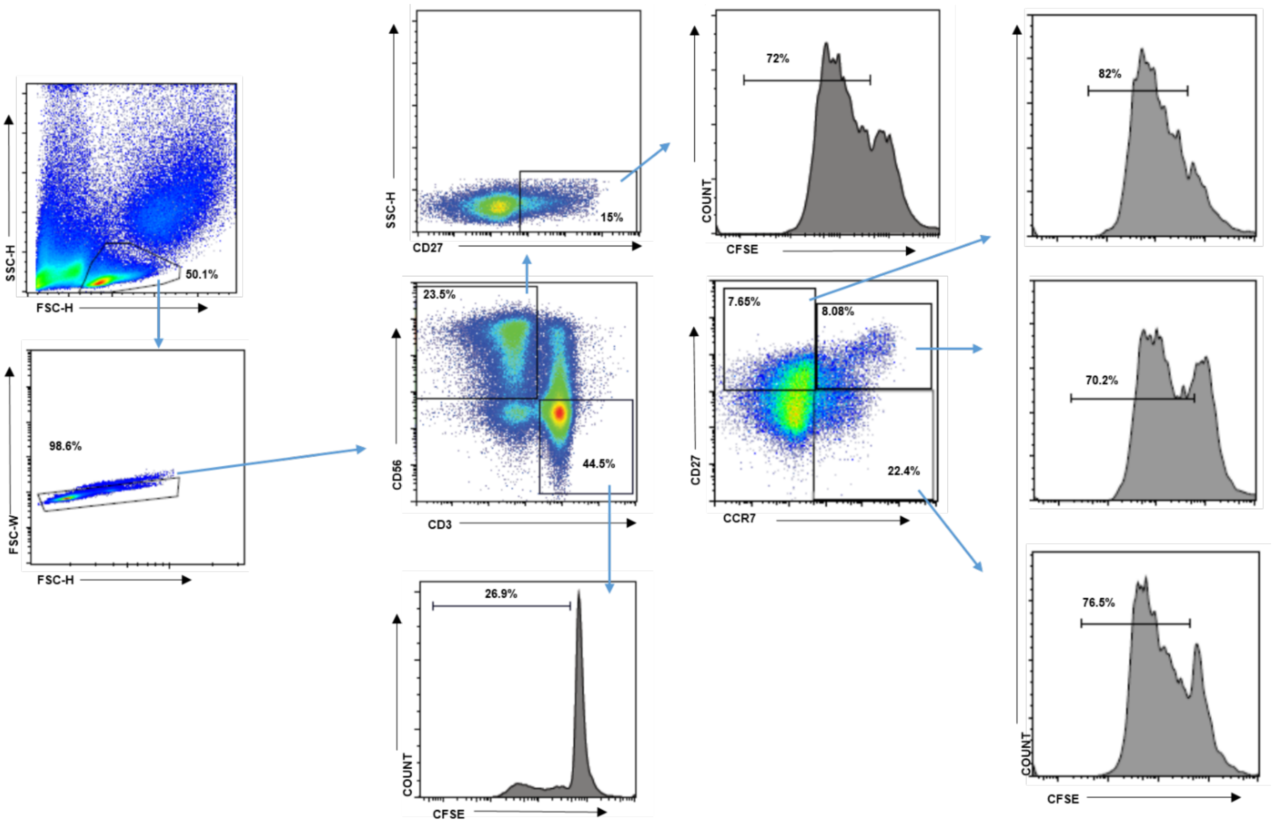

Figure S20 Gating strategy for the proliferation of memory-like NK cells. PBMCs from LTBI+ donors (n = 6) were labeled with CFSE and cultured with or without γ-Mtb. Some wells were supplemented with metabolites that were highly enriched in the plasma of nonconverters (4-hydroxypyridine, DL-methionine sulfoxide, L-kynurenine, L-α-glycerophosphocholine, D-sedoheptulose 7-phosphate, deoxycorticosterone acetate, each 100 µM). After 5 days, the proliferation of memory NK cells was measured by flow cytometry. A representative flow cytometry plot of the gating strategies is shown.

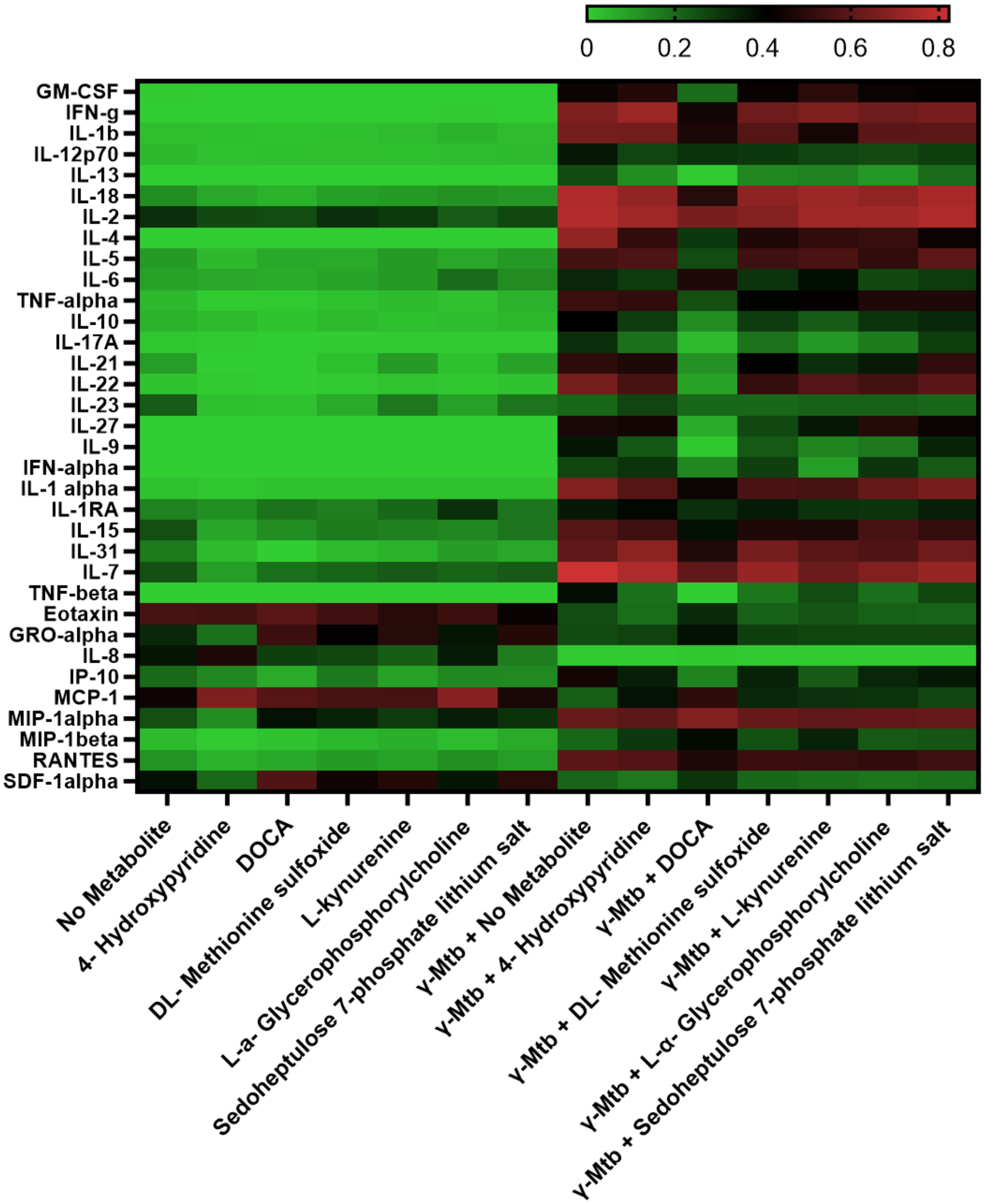

Figure S21 Cytokine and chemokine production by γ-Mtb-cultured MDMs in response to metabolites. Freshly isolated PBMCs from LTBI+ donors (n = 6) were cultured with or without γ-Mtb. Some of the γ-Mtb cultured cells were supplemented with various metabolites (4-

hydroxypyridine, DL-methionine sulfoxide, L-kynurenine, L-α-glycerophosphocholine, D-sedoheptulose 7-phosphate, deoxycorticosterone acetate). After 5 days, the cytokine and chemokine levels of the culture supernatants were determined by a multiplex ELISA (34 plex). A representative heatmap is shown.

Supplemental Table 1.

**Cohort Description**	
Total screened population	990
Percentage of existing LTBI+ individuals	538 (54.3%)
Total LTBI- cohort size for the current study	452 (45.6%)
Lost to follow-up	63 (13.9%)
Age range (Min-Max)	6 years – 73 years
Mean age in years	29
Percentage of males	51.6
Percentage of Females	48.4
Percentage of HIV positive	None
Percentage of pregnant women	None
Percentage of smokers	8
Percentage of alcohol consumers	11
Type of TB in the household	Pulmonary
Minimum months of exposure to the index case	3
Percentage of HHCs positive to IFN-γ release test	538 (54.3%)
Percentage of BCG vaccination	62
Percentage of city dwellers	92.1
Percentage of educated	60
Mean years of education	10
Average BMI	22.2

Supplemental Table 2.

Gene name	Forward sequence	Reverse sequence
Siglec-14	TCACCTGTCAGGTGAAACGCCA	ATGCTGATGGCGAGGTTCTGTG
Siglec-5	CTCACCTGTCAGATGAAACGCC	CCGTTCCTGAAGATGGTGATGG
Carboxylesterase 1 (CES1)	ATCCACTCTCCGAAGGGCAACT	GACAGTGTCGTCTGTTCCTCCT
RPS26	GGACATTTCTGAAGCGAGCGTC	CGATTCCTGACTACTTTGCTGTG
Annexin A1 (ANXA1)	GCGAAACAATGCACAGCGTCAAC	CAACCTCCTCAAGGTGACCTGT
RGCC	TCTCTGCCACTGTCACTCCTCA	GATGAAAGGACCCAGAACTTCTTG
Cathepsin K	GAGGCTTCTCTTGGTGTCCATAC	TTACTGCGGGAATGAGACAGGG
BD2 (DEFB4A)	ATAGGCGATCCTGTTACCTGCC	CATCAGCCACAGCAGCTTCTTG
Cathepsin B	GCTTCGATGCACGGGAACAATG	CATTGGTGTGGATGCAGATCCG
Cathelicidin (CAMP)	GACACAGCAGTCACCAGAGGAT	TCACAACTGATGTCAAAGGAGCC
defensin 4 (DEFA4)	ACTCCAGGCAAGAGGTGATGAG	ATCTGCAAGAGCAGACCATGCC
Cathepsin S	TGGATCACCACTGGCATCTCTG	GCTCCAGGTTGTGAAGCATCAC
S100A12	CTCTAAGGGTGAGCTGAAGCAG	ACCTGTTCATCTTGATTAGCATCC
Cathepsin L	GGAAAATGGAGGTCTGGACTCG	GTGTCATTAGCCACAGCGAACTC
Cathepsin D	GCAAACTGCTGGACATCGCTTG	GCCATAGTGGATGTCAAACGAGG
UBE2CBP	TGCCAATCCTGCGGTGAAGTCA	GTCAGGATGACAACACCATTCTC
Lactoferrin (LTF)	GGCTACTTCACTGCCATCCAGA	ACTCCACTGGTTACACTTGCGC
Lysozyme C	ACTACAATGCTGGAGACAGAAGC	GCACAAGCTACAGCATCAGCGA
S100A9	GCACCCAGACACCCTGAACCA	TGTGTCCAGGTCCTCCATGATG
S100A8	ATGCCGTCTACAGGGATGACCT	AGAATGAGGAACTCCTGGAAGTTA
FCN1	TGGCTCTGTGGACTTCTATCGG	CCTCAAAGTCCACCAGGTCTAC
HBD3	TTATTGCAGAGTCAGAGGCGGC	CTTTCTTCGGCAGCATTTTCGGC
FAU	CGTGCTGGAAAAGTGAGAGGTC	TGCCAAAGGTGGGCACAACGTT
Sig-5	CCCTCCCTTGGAAGAACAAA	TTAGGCTCCCTCGACTTCA
HBD1	GGTAACTTTCTCACAGGCCTTGG	TCCCTCTGTAACAGGTGCCTTG
ACTB	CACCATTGGCAATGAGCGGTTC	AGGTCTTTGCGGATGTCCACGT
HU-ADGRE1	TGTGACGTTGGACTTGGTAGCC	GGAGACAAAAGCCACACCAGTG
HU-CCR2 F	CAGGTGACAGAGACTCTTGGGA	GGCAATCCTACAGCCAAGAGCT
HU-CD14 F	CTGGAACAGGTGCCTAAAGGAC	GTCCAGTGTCAGGTTATCCACC
HU-CD68 F	CGAGCATCATTCTTTCACCAGCT	ATGAGAGGCAGCAAGATGGACC
HU-CSF1R F	GCTGCCTTACAACGAGAAGTGG	CATCCTCCTTGCCCAGACCAAA
HU-MARCO F	GGACAATTTGCGATGACGAGTGG	CCGACACTGAACATTATCCAGCC
HU-MRC1 F	AGCCAACACCAGCTCCTCAAGA	CAAAACGCTCGCGCATTGTCCA
HU-iNOS F	GCTCTACACCTCCAATGTGACC	CTGCCGAGATTTGAGCCTCATG
HU-PPAR gamma F	AGCCTGCGAAAGCCTTTTGGTG	GGCTTCACATTCAGCAAACCTGG
HU-SIGLEC1 F	ACCTGGAGGAAACTGACAGTGG	CTCAGTGTCACTGCCTGTCCTT
HU-TLR2 F	CTTCACTCAGGAGCAGCAAGCA	ACACCAGTGCTGTCCTGTGACA
HU-ARG1 F	TCATCTGGGTGGATGCTCACAC	GAGAATCCTGGCACATCGGGAA
HU-CD163 F	CCAGAAGGAACTTGTAGCCACAG	CAGGCACCAAGCGTTTTGAGCT
HU-CD200R1 F	CGTCTCCCATTTGACTGGCAAC	CCAAATGAATCCCACGATGGTCA
HU-CD80 F	CTCTTGGTGCTGGCTGGTCTTT	GCCAGTAGATGCGAGTTTGTGC
HU-CD86 F	CCATCAGCTTGTCTGTTTCATTCC	GCTGTAATCCAAGGAATGTGGTC

HU-CLEC10A F	GGTGGATGGAACAGACTATGCG	GGATGGAAGTGAGCACAGTCCT
HU-CLEC7A F	ACAATGCTGGCAACTGGGCTCT	AGAGCCATGGTACCTCAGTCTG
HU-CSF2 F	GGAGCATGTGAATGCCATCCAG	CTGGAGGTCAAACATTTCTGAGAT
HU-CXCR1 F	TCCTTTTCCGCCAGGCTTACCA	GGCACGATGAAGCCAAAGGTGT
HU-FCGR1A F	ATACAGGTGCCAGAGAGGTCTC	CCAGCTTATCCTTCCACGCATG
HU-ITGAM F	GGAACGCCATTGTCTGCTTTCG	ATGCTGAGGTCATCCTGGCAGA
HU-MERTK F	CAGGAAGATGGGACCTCTCTGA	GGCTGAAGTCTTTCATGCACGC
HU-PDCD1LG2 F	CTCGTTCCACATACCTCAAGTCC	CTGGAACCTTTAGGATGTGAGTG
HU-TNF F	CTCTTCTGCCTGCTGCACTTTG	ATGGGCTACAGGCTTGTCACTC
HU-CCL22 F	TCCTGGGTTCAAGCGATTCTCC	GTCAGGAGTTCAAGACCAGCCT
HU-CD36 F	CAGGTCAACCTATTGGTCAAGCC	GCCTTCTCATCACCAATGGTCC
HU-CD40 F	CCTGTTTGCCATCCTCTTGGTG	AGCAGTGTTGGAGCCAGGAAGA
HU-IL10 F	TCTCCGAGATGCCTTCAGCAGA	TCAGACAAGGCTTGGCAACCCA
HU-IL1B F	CCACAGACCTTCCAGGAGAATG	GTGCAGTTCAGTGATCGTACAGG
HU-IL6 F	AGACAGCCACTCACCTCTTCAG	TTCTGCCAGTGCCTCTTTGCTG
HU-LGALS3 F	CCATCTTCTGGACAGCCAAGTG	TATCAGCATGCGAGGCACCACT
HU-MYH14 F	CAGCCGTCAAATGCAAACCGAG	TTGCCTCTGTCGTCACCTTCTC
HU-TLR4 F	CCCTGAGGCATTTAGGCAGCTA	AGGTAGAGAGGTGGCTTAGGCT
HU-CCL2 F	AGAATCACCAGCAGCAAGTGTCC	TCCTGAACCCACTTCTGCTTGG
HU-CCR5 F	TCTCTTCTGGGCTCCCTACAAC	CCAAGAGTCTCTGTCACCTGCA
HU-CD209 F	GCAGTCTTCCAGAAGTAACCGC	GCTCTCCTCTGTTCCAATACTGC
HU-CD63 F	CAACCACACTGCTTCGATCCTG	GACTCGGTTCTTCGACATGGAAG
HU-CD86 F	CCATCAGCTTGTCTGTTTCATTCC	GCTGTAATCCAAGGAATGTGGTC
HU-CSF1 F	TGAGACACCTCTCCAGTTGCTG	GCAATCAGGCTTGGTCACCACA
HU-CXCL2 F	GGCAGAAAGCTTGTCTCAACCC	CTCCTTCAGGAACAGCCACCAA
HU-FCGR3A F	GGTGACTTGTCCACTCCAGTGT	ACCATTGAGGCTCCAGGAACAC
HU-IFNG F	GAGTGTGGAGACCATCAAGGAAG	TGCTTTGCGTTGGACATTCAAGTC
HU-IL4 F	CCGTAACAGACATCTTTGCTGCC	GAGTGTCCTTCTCATGGTGGCT
HU-IRF4 F	GAACGAGGAGAAGAGCATCTTCC	GAACGAGGAGAAGAGCATCTTCC
HU-ITGAX F	GATGCTCAGAGATACTTCACGGC	CCACACCATCACTTCTGCGTTC
HU-MSR1 F	TGCACAAGGCAGCTCACTTTGG	GTGCAAGTGACTCCAGCATCTTC
HU-PDGFB F	GAGATGCTGAGTGACCACTCGA	GTCATGTTCAGGTCCAACTCGG
HU-PTPRC F	CTTCAGTGGTCCCATTGTGGTG	CCACTTTGTTCTCGGCTTCCAG
HU-STAT6 F	CCTTGGAGAACAGCATTCCTGG	GCACTTCTCCTCTGTGACAGAC
HU-CLEC6A F	TCAGTGAAGGGACAAAGGTGCC	CTCCCATCTCAACACAGTTCTGC
HU-IL1R1 F	GTGCTTTGGTACAGGGATTCCTG	CACAGTCAGAGGTAGACCCTTC
HU-ITGB2 F	AGTCACCTACGACTCCTTCTGC	CAAACGACTGCTCCTGGATGCA
HU-PDCD1LG2 F	CTCGTTCCACATACCTCAAGTCC	CTGGAACCTTTAGGATGTGAGTG
HU-TLR7 F	CTTTGGACCTCAGCCACAACCA	CGCAACTGGAAGGCATCTTGTAG

SUPPLEMENTAL METHODS

Isolation and culture of PBMCs.

PBMCs were isolated by density gradient centrifugation using Ficoll-Hypaque. Whole blood diluted with RPMI was layered over an equal volume of Ficoll and centrifuged for 30-40 min at 2000 RPM without braking. PBMCs were washed twice and counted by trypan blue staining. Freshly isolated PBMCs were cultured in 24-well plates at 2×10^6 cells/well in RPMI 1640 containing 1% penicillin/streptomycin (Sigma), L-glutamine and 10% heat-inactivated human serum with or without CFP-10 + ESAT-6 (10 μg/ml) or γ-irradiated Mtb at 37 °C in a humidified atmosphere with 5% CO_2. After 96 h, the cell-free culture supernatants were collected, aliquoted and stored at -80 °C until the cytokine concentrations were measured by ELISA according to the manufacturer's guidelines.

Antigens for stimulation assays.

For stimulation of the PBMCs, we used ESAT-6 and CFP-10 peptide pools (BEI resources, USA) that consisted of 21 and 22 peptides covering the entire 6-kDa ESAT-6 and 10-kDa CFP-10, respectively. For some experiments, monocytes were cultured with γ-irradiated Mtb (γ-Mtb) (BEI resources, USA).

In vitro proliferation.

PBMCs were isolated by differential centrifugation over Ficoll-Paque (Amersham Pharmacia Biotech, Pittsburgh, PA, USA) and stained with carboxyfluorescein succinimidyl ester (CFSE) (5 μM) dye. Stained PBMCs were cultured in 12-well plates at 2×10^6 cells per well in RPMI-1640 containing 10% heat-inactivated human serum and γ-Mtb at 37 °C. In some experiments, cells were cultured in the presence of various metabolites. After 5 days, the expansion of

CD3−CD56+CD27+, CD3−CD56+CD27−, CD3−CD56+CD27+CCR7+, and CD3−CD56+CD27+CCR7- cells was determined by immunolabeling and flow cytometry.

Antibodies and other reagents.

PE anti-CD16, PerCP anti-CD14, and APC anti-CD56 were used to identify monocytes and NK cells. FITC anti-CCR7, PE anti-CD27, PERCP anti-CD3, and APC anti-CD56 were used to identify the T and NK cell populations. FITC anti-CD4, PE anti-Foxp3, and APC anti-CD25 were used for T regulatory cells (all antibodies used were from BD Bioscience, USA). Furthermore, we used PE-conjugated anti-human TNF-α and APC/Cy7-conjugated anti-human CD4 antibodies. In some experiments, Mtb-infected MDMs were stained with Alexa Fluor 488 anti-LC3 antibody (Millipore Inc.).

Flow cytometry.

Briefly, a total of 1×10^6 PBMCs were stained for three cell subsets. The T cell, monocyte, and NK cell subsets were surface stained with the respective antibodies and incubated in the dark for 30 min before being analyzed by flow cytometry. To determine the FoxP3 population, we used an intracellular staining kit from eBioscience. For surface staining, CD4 and CD25 were added to the cells before they were permeabilized. Anti-FoxP3 antibody was then added to the cells resuspended in staining buffer. After incubation, the cells were washed twice and fixed in 1% paraformaldehyde before acquisition on a FACSCalibur (BD Biosciences).

Measurement of cytokine production.

The ESAT-6- and CFP-10- or γ-Mtb-stimulated PBMCs as well as the various metabolite-treated MDM culture supernatants were stored at -70 °C until the cytokine concentrations were measured.

In the culture supernatants, the following 34 cytokines (34-Plex Human ProcartaPlex™ Panel 1A, Thermo Fisher Scientific) and chemokines or 20 cytokine chemokines (R&D Systems) were measured using a multiplex ELISA kit (as per the manufacturer's instructions: eotaxin/CCL11; GM-CSF; GRO alpha/CXCL1; IFN alpha; IFN gamma; IL-1 beta; IL-1 alpha; IL-1RA; IL-2; IL-4; IL-5; IL-6; IL-7; IL-8/CXCL8; IL-9; IL-10; IL-12 p70; IL-13; IL-15; IL-17A; IL-18; IL-21; IL-22; IL-23; IL-27; IL-31; IP-10/CXCL10; MCP-1/CCL2; MIP-1 alpha/CCL3; MIP-1 beta/CCL4; RANTES/CCL5; SDF1 alpha/CXCL12; TNF alpha; and TNF beta/LTA). The assay was run in batches by an independent reader who was blinded to the specimen status.

Measurement of antimicrobial peptide production.

γ-Mtb stimulated MDM culture supernatants were stored at -70 °C until the antimicrobial peptide concentrations were measured. In the culture supernatants, the level of LL-37 (HK321-02), defensin beta 1 (NBP2-77363), defensin beta 2 (NBP2-67933) and S1008/9 (DS8900) were measured using a multiplex ELISA kit (as per the manufacturer's instructions).

Preparation of the total RNA library and sequencing.

Total RNA from unstimulated and ESAT-6+CFP-10-stimulated PBMCs of nonconverters and converters was extracted with TRIzol reagent according to the manufacturer's instructions. The RNA was quantified and assessed for purity using a 2100 Bioanalyzer (Agilent Technologies, Waldbronn, Germany). A sequencing library was prepared with 1 μg of total RNA for each sample using the Illumina TruSeq™ RNA Sample Preparation Kit (Illumina, San Diego, CA, USA). In brief, the total RNA sample was treated with the Ribo-Zero rRNA Removal Kit (Epicenter, Madison, WI, USA) to deplete bacterial and eukaryotic ribosomal RNA (rRNA). The remaining

RNA was converted to single-stranded cDNA using reverse transcriptase and random hexamers, and the second strand was synthesized to produce double-stranded cDNA ready for application to TruSeq library construction using DNA Polymerase I and RNase H. These cDNA fragments underwent an end repair process, the addition of a single 'A' base, and ligation of the indexing adapters. The products were purified and enriched with PCR to create the final cDNA library. Finally, the libraries were quantified and qualified using a qPCR quantification protocol guide (KAPA Library Quantification Kits for Illumina Sequencing platforms) and TapeStation D1000 ScreenTape (Agilent Technologies), respectively. The resulting cDNA libraries were sequenced using the HiSeq2500 platform (Illumina), which generated approximately 453 million paired-end reads of 101 nucleotides in length.

Transcriptome and bioinformatics analysis.

Methods for data normalization and analysis are based on the use of "internal standards" that characterize some aspects of the system's behavior, such as technical variability, as presented elsewhere (1, 2). Created initially for the analysis of microarray data, these standards were slightly modified to be applicable to the RNA-seq data analysis. Differential gene expression analysis included the following steps. 1. Construction of the 'reference group' by identifying a group of genes expressed above background with inherently low variability as determined by an F test. The 'reference group' presents an internal standard of equal expression. As such, the 'reference group' is used to assess the inherent variability resulting from technical factors alone (technological variation). By creating an estimate of the technological variation, we were able to select a group of biologically stable genes. 2. Replications were selected using the commonly accepted significance threshold of $p<0.05$ with the Student's t test. This selection maintains the commonly accepted sensitivity level; however, a significant proportion of genes identified as differentially

expressed at this threshold will represent false-positive determinations. 3. An associative T test in which the replicated residuals for each gene from the experimental group are compared with the entire set of residuals from the reference group defined above. The Ho hypothesis was checked to determine whether the levels of gene expression in the experimental group presented as replicated residuals (deviations from the averaged control group profile) were associated with a highly representative (several hundred members) normally distributed set of residuals of gene expression values in the reference group. The significance threshold was then corrected to render the appearance of false-positive determinations improbable: in the current case, the threshold was set at $p<0.0001$. Only genes that passed both tests were presented in the final selections. Additional restrictions were applied to the minimal gene expression level (RPKM>2) and fold change (>1.5). The two-step normalization procedure and the associated analysis functions were implemented in MATLAB (Mathworks, MA). Functional analysis of identified genes was performed based on an Ingenuity Pathway Analysis (IPA; Ingenuity® Systems, Redwood City, CA, http://www.ingenuity.com). Data availability. The raw fastq files, analyzed files, and metadata file were deposited in the NCBI's Gene Expression Omnibus database (GEO GSE211566).

Metabolome Extraction and Liquid Chromatography–Mass Spectrometry. Plasma samples were collected from nonconverters and converters at baseline (0 months) and follow-up (24 months) and shipped to the metabolomics core facility at the Children's Medical Center Research Institute at UT Southwestern (Dallas, TX) for liquid chromatography–mass spectrometry (LC–MS). In brief, LC–MS was performed in MRM mode on a triple quadrupole mass spectrometer with two different dilutions of sample plus four retention times and three quality control standards.

Metabolome data analysis

Metabolites were log-transformed and autoscaled. Principal component analysis (PCA) and two-dimensional partial least squares discriminant analysis (2-D PLS-DA) score plots were used to compare plasma metabolite data across and between study groups; 2000-fold permutation tests were used to minimize the possibility that the observed separation of the PLS-DA was due to chance. Coefficient scores and the least absolute shrinkage and selection operator (LASSO) algorithm were used to identify the most discriminating metabolites for group comparisons. Heatmaps were generated using metabolites that were significantly different across the groups ($p<0.05$). Metabolite data analyses were performed using MetaboAnalyst.

Metabolite concentration determination and cytotoxicity assay.

Each metabolite was tested for its cytotoxicity against macrophages using a 3-(4,5-dimethylthiazol-2-yl)-2,5-diphenyl tetrazolium bromide (MTT) assay (various concentrations ranging from 125 μg/ml to 1 mg/ml). Metabolite concentrations were selected based on their cytotoxicity and the physiological concentration present in the plasma of healthy individuals (100% cells were viable until 72 hours).

Real-time PCR

Total RNA was extracted from whole PBMCs or MDMs using TRIzol (Invitrogen) according to the manufacturer's instructions. RNA was reverse transcribed (iScriptTM Reverse Transcription Supermix for RT–qPCR), and real-time PCR was performed using iTaqTM Universal SYBR® Green Supermix (BIO-RAD) according to the manufacturer's instructions. Gene expression analysis was performed in a QuantStudio 7 Flex (Applied Biosystems). All gene expression levels were normalized to β-actin/GAPDH internal controls in each sample, and the fold changes were

calculated using the 2-ΔΔCT method. The primers used in this study are listed in Supplemental Table 2.

Small interfering RNA.

Freshly isolated MDMs were transfected with small interfering RNA (siRNA) targeting CES-1, Siglec-14, RPS-26, RGCC-1, and ANX1 or with control siRNA using transfection reagents (all from Santa Cruz Biotechnology). In brief, 3 x 10^6 MDMs were resuspended in 500 µl of transfection medium and transfected with siRNA (6 pmol). After 6 h, an additional 500 µl of 2X RPMI-1640 complete medium was added, and the cells were cultured overnight in a 12-well plate. The next day, MDMs were washed and infected with Mtb H37Rv as outlined above, and after 5 days, the CFU counts were determined.

Isolation of monocytes by magnetic activated cell sorting.

Human blood-derived monocytes were sorted using anti-human CD14 MicroBeads (Miltenyi Biotec, Bergisch Gladbach, Germany) according to the manufacturer's protocol. Similarly, monocytes were isolated by negative sorting using the monocyte isolation Kit II (Miltenyi Biotec, Bergisch Gladbach; Germany) according to the manufacturer's protocol.

Isolation of Siglec-14+ monocytes.

Monocytes were isolated by the classical monocyte isolation kit (untouched negative selection) (Miltenyi Biotec, Bergisch Gladbach; Germany) from PBMCs. From the negatively selected CD14+ cells (>90% CD14+), Siglec-14+ cells were isolated by positive isolation using APC-conjugated anti-human Siglec-5/Siglec-14 antibody and anti-APC microbeads (Miltenyi Biotec, Bergisch Gladbach; Germany) according to the manufacturer's instructions. The purity of the isolated cells was >93%, as determined by flow cytometry.

Determination of Mtb H37Rv growth in human monocyte-derived macrophages (MDMs).

CD14+ monocytes (0.5×10^6 cells/well) were plated in 24-well plates in 1 ml of antibiotic-free macrophage serum-free medium (SFM) (Gibco, Gaithersburg, MD) and incubated at 37 °C in a humidified 5% CO2 atmosphere for up to 6 days. The culture media was renewed every day, and the cells were monitored morphologically for differentiation. After 6 days (after differentiation of the monocytes into macrophages), MDMs were transfected with small interfering RNA (siRNA) targeting CES1, SIGLEC-14, RPS26, ANX1 and RGCC1 or with control siRNA using transfection reagents (Santa Cruz Biotechnology). The efficiency of siRNA knockdown was measured by real-time PCR. In brief, MDMs were resuspended in 500 µl of transfection medium and transfected with siRNA (6 pmol). After 6 h, an additional 500 µl of 2X RPMI-1640 complete medium was added, and the cells were cultured overnight. The next day, the cells were washed and infected with *Mtb* H37Rv at an MOI of 1:2.5 (one MDM to 2.5 Mtb), incubated at 37 °C for 2 h, washed to remove extracellular bacilli, and cultured in macrophage-SFM (Gibco, Gaithersburg, MD). The cells were cultured for 120 h. The supernatant was aspirated, and the MDMs were lysed. The supernatant was centrifuged to pellet the bacteria, and the pellets were added to the cell lysates. The bacterial suspensions were ultrasonically dispersed, serially diluted, and plated in triplicate on 7H10 agar. The number of colonies was counted after 3 weeks.

Apo-Direct TUNEL assay.

Apoptosis was confirmed by the determination of single DNA strand breaks using an Apo-Direct apoptosis detection kit (Invitrogen). In brief, Siglec-14- or control siRNA-transfected macrophages were infected with Mtb H37Rv at an MOI of 1:2.5 (2.5 Mtb to one MDM). After 72 h, macrophages were harvested using PBS-EDTA, washed twice in PBS and fixed in 1% paraformaldehyde and 70% cold ethanol in PBS. After an overnight incubation at -20 °C, the cells

were washed in washing buffer, resuspended in 50 µl of DNA labeling solution (provided in the kit) and incubated at 37 °C. After 60 min, the cells were washed twice in rinsing buffer, and 500 µl of propidium iodide/RNase A solution was added and incubated in the dark. After 30 min, the cells were analyzed by FACS.

In vitro opsonization-mediated cell death.

Mtb H37Rv (5 $\times 10^7$ CFU) was incubated (37 °C, 1 h) with heat-inactivated serum (40 µl) collected at baseline (0 months) from converters and nonconverters. After sonication, Mtb H37Rv was washed with PBS and suspended in RPMI 1640 medium to infect freshly prepared MDMs at an MOI of 2.5 for 72 h at 37 °C. TUNEL staining was performed as described in the above Apo-Direct TUNEL assay to determine the opsonized Mtb-induced cell death of macrophages (3).

Autophagy detection assay.

Autophagy was detected using an LC-3 antibody-based assay kit (Millipore's FlowCellect) according to the manufacturer's instructions. In brief, MDMs were infected with Mtb H37Rv at an MOI of 2.5. After 72 h, macrophages were harvested using PBS-EDTA and washed once in 1X assay buffer (provided in the kit) followed by centrifugation at 300 g for 5 min. The pellet was suspended in 1X autophagy reagent B and immediately centrifuged at 300 g for 5 min. The supernatant was aspirated, and the pellet was again resuspended in 95 µl of 1X assay buffer + 5 µl of 20X anti-LC-3/FITC antibody for 30 min at room temperature in the dark. The cells were washed once with 1X assay buffer to remove unbound antibody followed by centrifugation at 300 g for 5 min and then resuspended in 1X assay buffer, and then the data were acquired by FACS.

Mitochondrial stress test assay.

MDMs were freshly prepared from the PBMCs of LTBI- nonexposed healthy donors and plated in XF96 plates. The mito-stress test (Agilent Technologies; Cat: 103015-100) was performed on a Seahorse Xfe96 Analyzer (Agilent, Santa Clara, CA). Cells were cultured in XF RPMI medium, pH 7.4 (Agilent Technologies; Cat: 103576-100) supplemented with 1 mM pyruvate, 2 mM glutamine and 10 mM glucose before measurement. Measurement of OCR (oxygen consumption rate), ECAR (extracellular acidification rate) and PER (proton efflux rate) was performed with subsequent injections of 0.66 μM oligomycin, 1 μM FCCP (carbonyl cyanide-4 trifluoromethoxy phenylhydrazone) and 0.5 μM rotenone/antimycin A. Spare respiratory capacity was measured as the maximum OCR after FCCP injection subtracted from the basal OCR under steady state conditions. The coupling efficiency was defined as the ratio between ATP-coupled respiration and proton leakage after oligomycin injection. GlycoATP and MitoATP production were calculated considering the proton efflux rate (PER), as extracellular acidification was a combined effect of both CO2 produced by OXPHOS and H+ ions produced by lactate formed due to glycolysis through the production of lactate. The following formula was used by the Wave analysis software to calculate the following:

glycoATP Production Rate (pmol ATP/min) = glycoPER (pmol H+/min)

mito ATP production Rate (pmol ATP/min) = OCRATP (pmol O2/min)*2 (pmol O/pmol O2)*P/O(pmol ATP/pmol O). Wave Desktop 2.6 software (Agilent) was used for the data analysis (4).

Supplemental Method References:

1. Dozmorov I, Lefkovits I. Internal standard-based analysis of microarray data. Part 1: analysis of differential gene expressions. Nucleic Acids Research. 2009;37(19):6323-39.
2. Dozmorov I, Centola M. An associative analysis of gene expression array data. Bioinformatics. 2003;19(2):204-11.
3. Lu LL, Chung AW, Rosebrock TR, Ghebremichael M, Yu WH, Grace PS, et al. A Functional Role for Antibodies in Tuberculosis. Cell. 2016;167(2):433-43.e14.
4. Zhou B, Magana L, Hong Z, Huang LS, Chakraborty S, Tsukasaki Y, et al. The angiocrine Rspondin3 instructs interstitial macrophage transition via metabolic–epigenetic reprogramming and resolves inflammatory injury. Nature Immunology. 2020;21(11):1430-43.
